# S2V-DREME: a time-resolved slice-to-volume MR image reconstruction framework with dynamic reconstruction and motion estimation

**DOI:** 10.1088/1361-6560/ae9d13

**Published:** 2026-09-03

**Authors:** Xiaoxue Qian, Hua-Chieh Shao, Jie Deng, Yan Dai, You Zhang

**Affiliations:** The Medical Artificial Intelligence and Automation (MAIA) Laboratory, Department of Radiation Oncology, University of Texas Southwestern Medical Center, Dallas, TX 75390, United States of America

**Keywords:** slice-to-volume reconstruction, motion tracking, time-resolved MRI, implicit neural representation

## Abstract

*Objective.* Existing volumetric magnetic resonance imaging (MRI) techniques are constrained by the trade-off between acquisition time and image quality, limiting accuracy in motion-impacted sites such as the liver. To enable fast, better-quality volumetric imaging with sufficient spatiotemporal resolution, we developed a time-resolved volumetric MRI technique that recovers 3D volumes from acquired 2D MR slices for real-time 3D anatomy and motion tracking. *Approach.* 2D MR slices dynamically acquired in time and space were mapped to time-resolved 3D MRIs using a one-shot slice-to-volume framework, S2V-DREME. The model jointly estimates a reference 3D MRI and time-resolved deformation vector fields (DVFs) that warp the reference volume into dynamic 3D MRIs. The reference volume is represented by a spatial implicit neural representation (INR), while the DVFs are derived via low-rank motion modeling. Motion basis components (MBCs) are generated by a spline-enhanced INR (SINR)-based motion generator, with coefficients inferred by a feature-wise linear modulation-based motion encoder. A progressive optimization strategy sequentially initializes the spatial INR and MBCs before joint optimization. The loss function integrates slice data fidelity, total variation regularization, MBC normalization, and DVF smoothness constraints. *Main results.* S2V-DREME generates time-resolved volumetric MRIs from 2D MR slice inputs. It was evaluated on digital phantom extended cardiac torso (XCAT), physical phantom, and human studies. In XCAT, it accurately captured regular and irregular motion during dynamic reconstruction (training stage, Dice similarity coefficient (DSC)/COME: 0.92 ± 0.03/0.98 ± 0.43 mm) and real-time motion estimation (testing stage, DSC/COME: 0.91 ± 0.02/0.99 ± 0.73 mm). Physical phantom experiments achieved a mean COME of 1.16 mm, and human studies demonstrated the feasibility of time-resolved 3D reconstruction from orthogonal-view and single-view slice acquisitions. *Significance.* By combining a novel step-and-shoot acquisition protocol with motion-compensated one-shot learning, S2V-DREME enables accurate time-resolved volumetric MRI reconstruction and motion tracking from cineslices, with strong potential for rapid volumetric imaging and real-time MR-guided adaptive radiotherapy.

## 1. Introduction

Benefiting from its high soft-tissue contrast, elimination of ionizing radiation, and capabilities to provide functional and biological characterization, magnetic resonance imaging (MRI) (Kuperman 2000, Katti *et al* 2011) has rapidly advanced and become an essential component of modern clinical radiotherapy workflows, playing a pivotal role in MRI-guided radiotherapy planning and delivery (Khoo *et al* 1997, Tsien *et al* 2014, Schmidt and Payne 2015). However, patient motion remains a fundamental challenge in MRI acquisition and reconstruction, resulting in motion-induced artifacts and temporal blurring. These motion-related degradations compromise image quality and anatomical fidelity, thereby increasing uncertainty in target localization and reducing the accuracy of radiation dose delivery (Dirix *et al* 2014, Liney and Moerland 2014). Such challenges are particularly pronounced in abdominal organs subject to continuous respiratory motion, such as the liver, where inaccurate motion estimation may ultimately compromise treatment precision, therapeutic efficacy, and overall clinical outcomes (Karlsson *et al* 2009, McBain *et al* 2009, Chandarana *et al* 2018).

Current state-of-the-art motion management strategies rely on 4D-MRI (Keall 2004, Remmert *et al* 2007, Li *et al* 2008) to characterize respiration-induced motion, including both prospective (Blackall *et al* 2006, Dinkel *et al* 2009) and retrospective motion-resolved imaging approaches (Cai *et al* 2011, Liu *et al* 2015). Prospective 4D-MRI incorporates respiratory information during data acquisition and is often realized using fast 3D MR sequences, typically enabled by acceleration techniques, for example, parallel imaging, echo-sharing, and k-space undersampling strategies. However, technical constraints impose inherent trade-offs between acquisition speed and image quality. Alternatively, retrospective 4D-MRI relies on continuous acquisition of MR signals across respiratory cycles and reconstructs motion-resolved volumetric images by retrospectively sorting the acquired data into predefined respiratory phase bins (Paganelli *et al* 2015, Deng *et al* 2016). This sorting-based strategy characterizes respiratory motion by averaging information over multiple breathing cycles and relies on the assumption of periodic and reproducible respiration. In real clinical settings, this assumption is frequently violated due to irregular breathing patterns, including variations in amplitude/frequency and the presence of baseline drift, which cause respiratory binning errors, motion blurring, and reduced volumetric fidelity (Lu *et al* 2005).

*Time-resolved dynamic MRI* (Gamper *et al* 2008, Lingala *et al* 2014, Li *et al* 2025) addresses the limitations of sorting-based 4D-MRI by leveraging the entire acquisition dataset for temporally correlated reconstruction to obtain continuous dynamic sequences. However, due to the complexity of spatiotemporal reconstruction and optimization, most time-resolved dynamic imaging techniques remain restricted to 2D formulations (Song and Dougherty 2004, Huang *et al* 2021), thereby limiting their applicability and potential for target tracking in radiotherapy. Radiotherapy demands volumetric imaging to monitor patient motion during treatment delivery, particularly in the thoracic and abdominal regions, where organ and tumor motion exhibits complex 3D patterns. Recently, a few studies tried to solve 3D time-resolved dynamic MR reconstruction based on a single MR scan, such as MR-MOTUS (Huttinga *et al* 2021a), Extreme MRI (Ong *et al* 2020), and STINR-MR (Shao *et al* 2024). They utilized temporal and spatial correlations across dynamic volumes to address the undersampling problem for each dynamic. However, these time-resolved dynamic MRI methods rely on a globally coupled reconstruction framework, which requires retrospective, joint processing of the entire acquisition set. These approaches do not apply to scenarios that require ultra-fast, time-resolved *real-time imaging* (Weiskopf *et al* 2007, Lombardo *et al* 2024). For instance, for real-time radiotherapy motion monitoring, dose accumulation, and treatment adaptation, real-time MR imaging with sub-second latency is required to capture 3D anatomical motion variations and assess the dose deviations to guide radiation delivery.

With the rapid development of deep neural networks, deep learning approaches have been explored for accelerated MRI reconstruction and real-time motion estimation. Existing methods were generally categorized into reconstruction-based and registration-based approaches. Reconstruction-based methods sought to recover high-quality images from heavily undersampled measurements by leveraging generative modeling (Shende *et al* 2019) or super-resolution techniques (Van Reeth *et al* 2012) to enhance spatial resolution and mitigate aliasing artifacts. However, due to the extreme undersampling challenge of 3D real-time imaging, many approaches are limited to a 2D setting (Yang *et al* 2017, Huang *et al* 2022), which constrained the model’s ability to capture rich spatial context, inter-slice continuity, and complex 3D anatomical structures. Registration-based approaches typically relied on a fully sampled reference volume and employed neural networks to estimate a deformation vector field (DVF) for motion tracking, where the estimation was constrained by a data-consistency loss between the warped reference and the undersampled target data (Terpstra *et al* 2021, Huttinga *et al* 2021b, Shao *et al* 2022). Some of these approaches adopted supervised training paradigms that required 3D motion fields as ground truth (GT), which were difficult to obtain in clinical practice. Moreover, population-based deep learning approaches usually rely on large training datasets and might suffer from limited generalizability and robustness when applied to out-of-distribution data. Qian *et al* introduced a hybrid framework (Qian *et al* 2025) combining population-level priors with patient-specific refinement to address the generalizability challenge, and later extended it using meta-learning (Qian *et al* 2026) to further accelerate test-time adaptation. Nevertheless, the test-time optimization (∼30 s) has yet to achieve real-time efficiency.

More recently, a new technique, DREME-MR (Shao *et al* 2025b), integrated *retrospective time-resolved dynamic MRI reconstruction* and *time-resolved real-time MR imaging* into a unified learning framework. Rather than training a population-based model, DREME-MR directly learns a patient-specific model from the to-be-reconstructed k-space measurements, yielding a ‘one-shot’ model immune to the generalizability challenges of conventional deep learning models. Specifically, from the raw k-space data of a pre-treatment MRI scan, DREME-MR learns a reference 3D MRI volume and a patient-specific motion model to describe the intra-scan dynamic motion. Additionally, the patient-specific motion model can utilize minimal k-space data (e.g. a few k-space spokes) acquired in the future, for instance during radiotherapy treatments, to directly infer real-time motion and corresponding real-time MRI volumes. Nevertheless, the k-space-driven DREME-MR needs repeated NUFFT operations (Fessler and Sutton 2003) during iterative optimization to handle non-Cartesian k-space trajectories for MRI reconstruction, incurring substantial computational cost during the reconstruction pipeline. In addition, extracting and handling raw k-space data within clinical workflows is generally nontrivial and requires additional preprocessing steps, making this technique not yet ready for routine clinical use. Real-time motion monitoring in current MR-LINAC systems is primarily performed using 2D cine imaging (Donahue *et al* 2025, Smith *et al* 2025), and adopting a k-space-based framework will require changes to the current clinical workflow.

To facilitate clinical deployment while maintaining high reconstruction efficiency and image quality for time-resolved MRI (both retrospective and real-time), we proposed S2V-DREME, a slice-to-volume dynamic reconstruction and motion estimation framework that reconstructed time-resolved 3D MR images from acquired 2D cine slices. Inheriting the same dynamic reconstruction and motion estimation idea from DREME-MR (Shao *et al* 2025b), S2V-DREME aims to develop a framework that can be more easily integrated into the current clinical workflow. By using 2D cine images as raw input, S2V-DREME was highly compatible with standard cine MRI acquisitions commonly used in motion monitoring/management of MR-guided radiotherapy workflows. Similar to DREME-MR, S2V-DREME jointly learned a patient-specific reference volume and time-resolved deformation fields in a ‘one-shot’ manner, thereby facilitating time-resolved volumetric reconstruction and real-time 3D motion tracking from sparse 2D observations. For effective slice-to-volume mapping, a ‘step-and-shoot’ acquisition scheme was proposed to acquire 2D cine data for S2V-DREME training. After training, S2V-DREME can use cine slices continuously acquired at fixed/desired locations (for instance, through the target center planes) for real-time motion estimation. To further improve efficiency, S2V-DREME incorporated a spline-enhanced implicit neural representation (SINR) (Sideri-Lampretsa *et al* 2024a) to generate motion basis components (MBCs) to accelerate model training. A feature-wise linear modulation (FiLM) (Perez *et al* 2018)-based motion encoder was designed to predict MBC scores and estimate motion from 2D slices together with their slice location information, enabling accurate estimation of patient-specific DVFs for dynamic reconstruction and motion estimation. Digital phantom simulations, physical phantom measurements, and volunteer scans were applied to evaluate S2V-DREME. We demonstrated the applicability of both single-view and orthogonal-view slice acquisition protocols for S2V-DREME reconstruction.

## 2. Materials and methods

**Figure 1.**
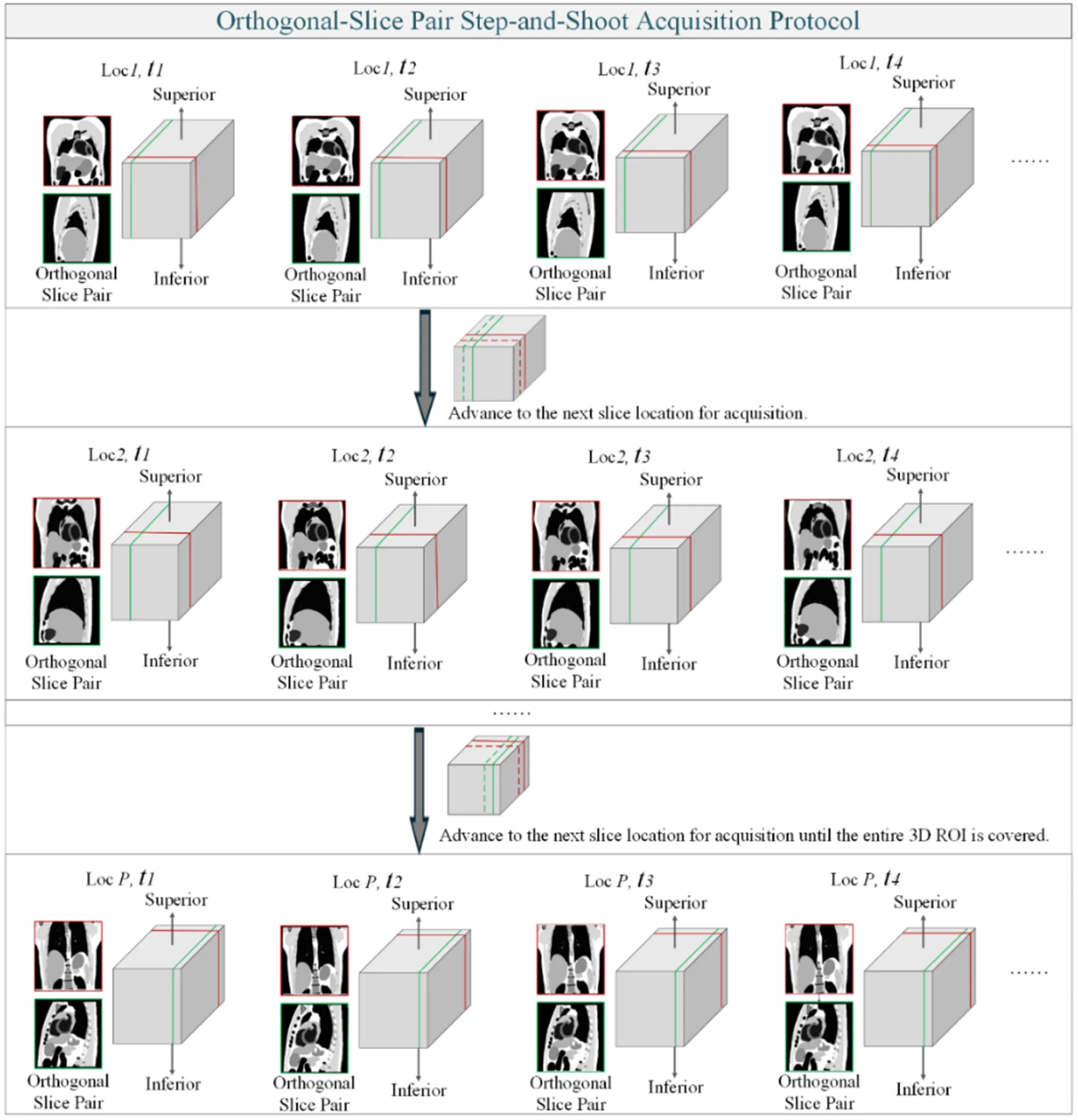
Step-and-shoot orthogonal-slice acquisition protocol for S2V-DREME.

**Figure 2.**
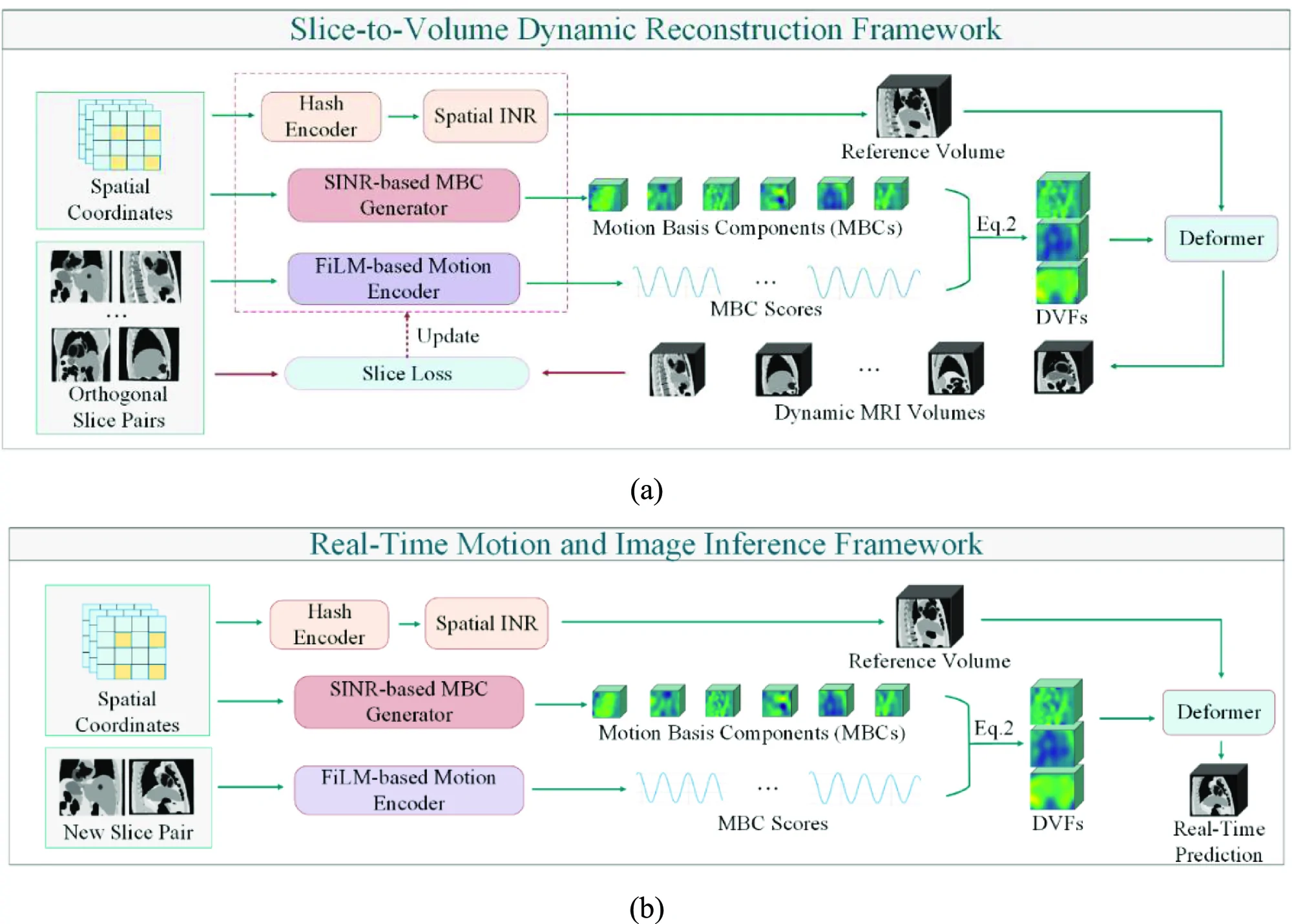
Reconstruction and inference framework of S2V-DREME: (a) slice-to-volume dynamic reconstruction and (b) real-time motion and image inference.

To achieve fast and reliable volumetric imaging with high spatiotemporal resolution, we developed the S2V-DREME framework, comprising three key components. First, as illustrated in figure 1, a ‘step-and-shoot’ orthogonal slice acquisition protocol was developed to progressively capture volumetric anatomical information through complementary orthogonal planes, providing spatial mapping and implicit motion tagging. Second, a patient-specific slice-to-volume dynamic reconstruction framework (figure 2(a)) jointly estimated a reference 3D MR image and a time-resolved motion model from the slice pairs within a unified optimization framework. Third, a real-time motion inference framework (figure 2(b)) enabled efficient estimation of time-varying motion states for real-time volumetric MRI reconstruction and 3D motion tracking during treatment.

### 2.1. Step-and-shoot MR slice acquisition protocol

To acquire motion-informative data while achieving desired 3D spatial mapping, we designed a step-and-shoot slice acquisition protocol, as illustrated in figure 1. Along the superior-inferior (SI) and anterior-posterior (AP) directions, at a slice location p, a pair of orthogonal 2D MRI slices (${{\boldsymbol{S}}_{\mathrm{sag}}},{\text{ }}{{\boldsymbol{S}}_{\mathrm{cor}}}$) were sequentially acquired in an interleaved fashion. Here, ${{\boldsymbol{S}}_{\mathrm{sag}}}$ and ${{\boldsymbol{S}}_{\mathrm{cor}}}$ denote the sagittal and coronal slices, respectively. Each slice pair can be acquired efficiently (<0.5 s) and viewed as under the same motion state/instance with the same imaging time tag t. The orthogonal slice pair was repeatedly sampled for *T* consecutive time frames to capture respiratory-induced temporal variations. After completing cine acquisition at location *p*, the slice position was incrementally advanced to the next slice location (p+1) along the two orthogonal directions. By acquiring a continuous cine sequence at each slice location before progressing to the next, temporal motion information at each slice location was captured concurrently with slice acquisition. This step-and-shoot process was repeated until all slice locations had been sampled, thereby progressively traversing the entire anatomy for volumetric sampling.

With this acquisition design, the step-and-shoot protocol enjoyed several practical advantages. By restricting data collection to sparse yet motion-informative 2D cine orthogonal slices at each instance, the acquisition burden was substantially reduced while essential motion information was preserved. Operating directly on image-domain slices, the protocol supported flexible pulse sequence design and allowed independent control over in-plane spatial resolution and temporal sampling. Importantly, the aggregation of these time-resolved orthogonal slice pairs across all slice locations provided sparse, motion-resolved coverage of the entire imaging volume, serving as the basis for subsequent time-resolved slice-to-volume reconstruction and motion modeling.

### 2.2. Slice-to-volume dynamic reconstruction framework

The slice-to-volume dynamic reconstruction (figure 2(a)) problem was formulated as a joint optimization over a static reference volume ${\hat {\boldsymbol{I}}_{\mathrm{ref}}}\left({\boldsymbol{v}} \right)$ and a time-resolved DVF $\hat {\boldsymbol{d}}\left( {{\boldsymbol{v}},t} \right)$, where $\hat \cdot $ denotes an estimated quantity. The estimated DVF warped the reference anatomy to produce time-resolved dynamic volumes $\hat{\boldsymbol{I}}\left( {{\boldsymbol{v}},t} \right)$, with reconstruction driven by a consistency loss between the reconstructed slices and the acquired orthogonal slices ${{\boldsymbol{S}}_{\mathrm{sag}}}\left( {{\boldsymbol{s}},{\text{ }}t} \right)$ and ${{\boldsymbol{S}}_{\mathrm{cor}}}\left( {{\boldsymbol{s}},{\text{ }}t} \right)$.

\begin{equation*}\hat{\boldsymbol{I}}\left( {{\boldsymbol{v}},t} \right) = {\hat {\boldsymbol{I}}_{\mathrm{ref}}}\left( {{\boldsymbol{v}} + \hat {\boldsymbol{d}}\left( {{\boldsymbol{v}},t} \right)} \right),\end{equation*}

where ${\boldsymbol{v}} \in {R^3}$ and t respectively denote the 3D voxel coordinates of the reconstruction volume and the frame index of the dynamic sequence. ${\text{ }}s \in {R^2}$ denotes 2D pixel coordinates on a slice.

The reference volume ${\widehat {\mathbf{I}}_{{\mathrm{ref}}}}\left( {\mathbf{v}} \right)$ was modeled using a hash-encoded spatial INR to improve learning efficiency and facilitate the representation of high-frequency anatomical details. Specifically, each queried 3D voxel coordinate was first passed through the hash encoder, which mapped the coordinate to multi-level feature vectors through hash-table lookup and trilinear interpolation (Müller *et al* 2022, Shao *et al* 2024). The feature vectors from all resolution levels were concatenated and then fed into the spatial INR. The spatial INR was implemented as a three-layer MLP with sinusoidal activation functions (SIREN) and 256 hidden units per layer to predict the corresponding voxel intensity of the reference volume from the hash-encoded spatial features.

In parallel, a low-rank motion model was introduced to characterize anatomical deformation using DVFs ${\boldsymbol{\hat d}}\left( {{\mathbf{v}},{\mathrm{t}}} \right)$. The DVFs were low-rank decomposed into products of MBCs $\left\{ {{{{\boldsymbol{\hat e}}}_{\mathrm{i}}}\left( {\mathbf{v}} \right)} \right\}_{{\mathrm{i}} = 1}^{\mathrm{L}}$ and their corresponding time-varying MBC scores $\left\{ {{{{\boldsymbol{\hat w}}}_{\mathrm{i}}}\left( {\mathrm{t}} \right)} \right\}_{{\mathrm{i}} = 1}^{\mathrm{L}}$:

\begin{equation*}\hat {\boldsymbol{d}}\left( {{\boldsymbol{v}},t} \right) = \mathop \sum \limits_{i = 1}^L {\hat {\boldsymbol{w}}_i}\left( t \right){\hat {\boldsymbol{e}}_i}\left( {\boldsymbol{v}} \right),\end{equation*}

where ${{\boldsymbol{\hat e}}_{\mathrm{i}}}\left( {\mathbf{v}} \right) \in {{\mathrm{R}}^{{\mathrm{L}} \times 3 \times {{\mathrm{N}}_{{\mathrm{voxel}}}}}}$, ${{\boldsymbol{\hat w}}_{\mathrm{i}}}\left( {\mathrm{t}} \right) \in {{\mathrm{R}}^{{\mathrm{L}} \times 3}}$, *L* denotes the number of levels in the low-rank decomposition. Three levels (L=3) were used for the spatial decomposition, which has been shown to be sufficient for representing respiratory motion (Li *et al* 2011).

In the previous DREME-MR framework (Shao *et al* 2025b), the MBCs were modeled by a learning-based B-spline interpolator (Tegunov and Cramer 2019), in which a set of learnable B-spline coefficients defined on a control grid was optimized and interpolated over all voxel coordinates to generate dense deformation fields. The corresponding MBC scores were predicted by an MLP-based motion encoder from the acquired k-space signals. The encoder was jointly trained during dynamic reconstruction to learn the mapping from k-space measurements to the time-varying motion-score vector. The time-resolved DVF ${\boldsymbol{\hat d}}\left( {{\mathbf{v}},{\mathrm{t}}} \right)$ was then computed as a weighted sum of the MBCs with their corresponding scores. To model the MBCs more efficiently, in this work, we employed a spline-enhanced INR (SINR) (Sideri-Lampretsa *et al* 2024a) as the MBC generator. It modeled the MBCs using a set of INRs and integrated them with B-spline free-form deformations (FFDs) (Rueckert *et al* 2002), enabling efficient and accurate modeling of anatomical motion. Each INR was trained to predict a displacement vector ${\hat {\boldsymbol{u}}_i}\left( {{{\boldsymbol{c}}_i}} \right)$ at a set of B-spline control point coordinates ${{\boldsymbol{c}}_i} = {\text{ }}{\left\{ {{c_1},{c_2}, \cdots ,{c_k}} \right\}_i}$ for the MBCs at the rank i, where the number of control points *k* was determined by the control-point spacing, which refers to the voxel interval between neighboring B-spline control points in each spatial dimension. The *ith*-rank MBCs ${\hat {\boldsymbol{e}}_i}\left({\boldsymbol{ v}} \right)$ were then obtained by B-spline FFD interpolation, as follows:

\begin{equation*}{\hat {\boldsymbol{e}}_i}\left({\boldsymbol{ v}} \right) = B\left( {{{\boldsymbol{c}}_i} + {{\hat {\boldsymbol{u}}}_i},{\boldsymbol{v}}} \right),\end{equation*}

where $B\left( { \cdot ,{\boldsymbol{v}}} \right)$ denotes the B-spline FFD interpolation evaluated at coordinates ${\boldsymbol{v}}$.

Compared with the original DREME-MR, which directly optimized the MBCs via voxel-wise B-spline interpolation, the SINR-based generator predicted control-point displacements using INRs and reconstructed dense MBC fields through B-spline FFD implemented as a convolution over the control grid, which improved GPU efficiency and reduced computational cost.

The MBC scores ${\hat {\boldsymbol{w}}_i}\left( t \right)$ were designed to characterize the temporal motion patterns captured from clinically acquired data. For S2V-DREME, the goal was to predict both the volumetric image and its associated motion from the acquired 2D slices. By explicitly indicating where the 2D slices were sampled within the 3D anatomical space during data acquisition, the slice locations provided strong spatial supervision for motion estimation. Since the motion/anatomical features of different slice locations differ, the slice location ${\text{ }}p$ serves as an index to condition the motion encoder to make location-aware inference, which facilitates robust learning of the motion encoder during its training. We incorporated the slice location information into the motion prediction process and proposed a FiLM-based motion encoder (Perez *et al* 2018) to predict the MBC scores ${\hat {\boldsymbol{w}}_i}\left( t \right)$ from the orthogonal slices ${{\boldsymbol{S}}_{\mathrm{sag}}}\left( {{\boldsymbol{s}},{\text{ }}t} \right),{\text{ }}{{\boldsymbol{S}}_{\mathrm{cor}}}\left( {{\boldsymbol{s}},{\text{ }}t} \right)$ and their slice location p.

For the orthogonal-view acquisition protocol, the motion encoder took two slices as input and output the corresponding MBC scores. Since different slice locations involve distinct anatomy and motion features, for S2V-DREME, we also incorporated the slice location information into the motion encoder to render accurate, spatial context-aware motion estimation. The slice location p was encoded using Fourier positional encoding $\phi \left( \cdot \right)$ and subsequently mapped by a fully connected MLP $f\left( \cdot \right)$ to generate a global modulation vector, which was then split into layer-wise FiLM parameters: $\{ {\gamma _c},{\beta _c}\} _{c = 1}^C = {\mathrm{Split}}\left( {f\left( {\phi \left( p \right)} \right)} \right)$. The modulation parameters ${\gamma _c}$ and ${\beta _c}$ were used to linearly modulate the convolutional features of the motion encoder. The FiLM operation was formulated as:

\begin{equation*}FiLM\left( {{{\boldsymbol{F}}_c}{\text{| }}{\gamma _c},{\text{ }}{\beta _c}} \right) = {\gamma _c}{{\boldsymbol{F}}_c} + {\beta _c},\end{equation*}

where c indexes the convolutional layers, C denotes the total number of layers, and ${{\boldsymbol{F}}_c}$ represents the feature map output of that layer.

**Figure 3.**
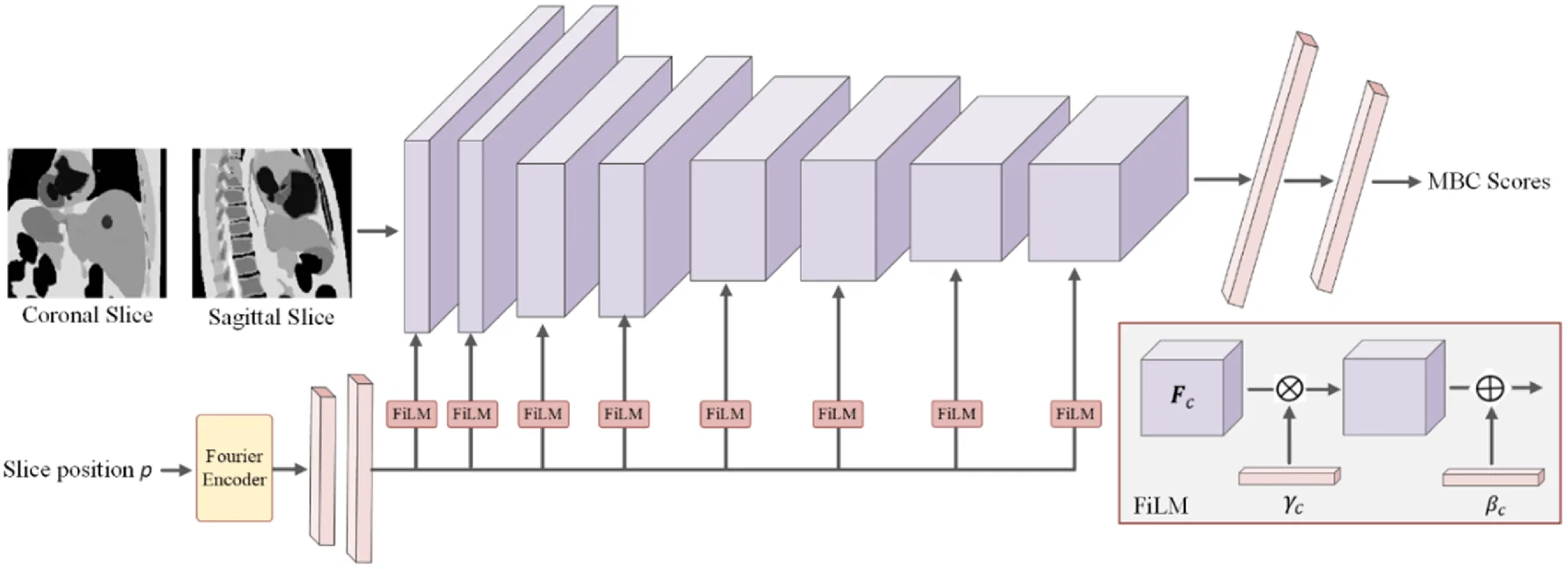
Overview of the FiLM-based motion encoder. Orthogonal slice pairs are processed by a convolutional backbone, while the slice location is encoded using Fourier positional encoding and mapped through fully connected layers to generate layer-wise FiLM parameters for modulating each convolutional block. The resulting features are then used to infer the MBC scores via fully connected layers.

The FiLM-based motion encoder was implemented as an eight-layer convolutional architecture, as illustrated in figure 3. It took an orthogonal slice pair and the corresponding slice position p as inputs. The two slices were first concatenated and fed into a convolutional backbone. The slice position was encoded using Fourier positional encoding and mapped by fully connected layers to generate layer-wise FiLM parameters $\{ {{{\gamma }}_{\mathrm{c}}},{{\beta }_{\mathrm{c}}}\} _{{\mathrm{c}} = 1}^{\mathrm{C}}$. These parameters were applied to the feature maps of each convolutional block through channel-wise affine modulation, enabling the extracted motion features to be conditioned on slice-position information. The final feature maps were flattened and passed through fully connected layers to infer the MBC scores.

### 2.3. Real-time motion inference framework

After dynamic reconstruction, S2V-DREME yielded a patient-specific reference volume, a set of MBCs learned by the SINR-based generator, and a FiLM-based motion encoder capable of estimating time-varying MBC scores for new orthogonal slice pairs. During real-time deployment for radiation treatment (figure 2(b)), orthogonal slice pairs can be acquired at a fixed location most relevant to the target under tracking (through the target center, for instance). For each slice pair, the motion encoder performed forward inference to estimate the corresponding MBC scores. These scores were then combined with the learned MBCs to compute DVFs, which were subsequently applied to the reference volume to generate real-time MR volumes. This design enabled low-latency volumetric imaging and continuous 4D motion tracking from orthogonal slice measurements.

### 2.4. Loss functions design and training procedures

Following the DREME-MR framework (Shao *et al* 2025b), we employed a similar, yet simpler, progressive two-stage optimization strategy for S2V-DREME due to the convenience of image-domain optimization. In stage I, the spatial INR and hash encoder (figure 2(a)) were trained for ${N_a}$ epochs to reconstruct the reference volume ${\hat {\boldsymbol{I}}_{\mathrm{ref}}}\left( v \right)$, yielding an initial anatomical representation to warm-start the reconstruction process. In stage II, the motion model, including the SINR-based MBC generator and FiLM-based motion encoder (figure 2(a)), was initially trained with the spatial INR and its hash encoder temporarily frozen. This design maintained training stability and prevented inaccurate motion estimates in the early stage from propagating to the learned reference volume. Afterward, all components were jointly optimized in an end-to-end manner.

In stage I, the spatial INR was optimized using a similarity loss of the mean squared error (MSE) between the reconstructed reference volume ${\hat {\boldsymbol{I}}_{\mathrm{ref}}}\left( {\boldsymbol{v}} \right)$ and an initial reference volume ${I_{\mathrm{ini}}}\left( {\boldsymbol{v}} \right)$:

\begin{equation*}\mathcal{L}_{\mathrm{sim}}^{ref} = \frac{1}{{{N_{\mathrm{voxel}}}}}\mathop \sum \limits_{i = 1}^{{N_{\mathrm{voxel}}}} {\left| {{{\hat {\boldsymbol{I}}}_{\mathrm{ref}}}\left( {{ {\boldsymbol{v}}_i}} \right) - {I_{\mathrm{ini}}}\left( {{ {\boldsymbol{v}}_i}} \right)} \right|^2},\end{equation*}

where ${N_{\mathrm{voxel}}}$ denotes the number of image voxels, and the initial reference volume ${{\boldsymbol{I}}_{\mathrm{ini}}}\left( {\boldsymbol{v}} \right)$ was obtained by averaging the acquired sagittal slices at each location and then stacking all locations together.

In stage II, joint optimization of the spatial INR and the motion model was driven by an MSE loss between the reconstructed slices ${\hat {\boldsymbol{S}}_{\mathrm{sag}}}\left( {{\boldsymbol{s}},\;t} \right),\;{\hat {\boldsymbol{S}}_{\mathrm{cor}}}\left( {{\boldsymbol{s}},\;t} \right)$ and the acquired orthogonal slices ${{\boldsymbol{S}}_{\mathrm{sag}}}\left( {{\boldsymbol{s}},{\text{ }}t} \right),{\text{ }}{{\boldsymbol{S}}_{\mathrm{cor}}}\left( {{\boldsymbol{s}},{\text{ }}t} \right)$:

\begin{equation*}\mathcal{L}_{\mathrm{sim}}^{slc} = \frac{1}{{{N_{batch}}}}\mathop \sum \limits_{t = 1}^{{N_{batch}}} \frac{1}{{{N_{pixel}}}}\mathop \sum \limits_{i = 1}^{{N_{pixel}}} \left( {{{\left| {{{\hat {\boldsymbol{S}}}_{\mathrm{sag}}}\left( {{s_i},\;t} \right) - {{\boldsymbol{S}}_{\mathrm{sag}}}\left( {{s_i},\;t} \right)} \right|}^2} + {{\left| {{{\hat {\boldsymbol{S}}}_{\mathrm{cor}}}\left( {{s_i},\;t} \right) - {{\boldsymbol{S}}_{\mathrm{cor}}}\left( {{s_i},\;t} \right)} \right|}^2}} \right),\end{equation*}

where the reconstructed slice ${\hat {\boldsymbol{S}}_{\mathrm{sag}}}\left( {{\boldsymbol{s}},{\text{ }}t} \right)$ is extracted from the time-resolved dynamic MR volume $\hat{\boldsymbol{I}}\left( { {\boldsymbol{v}},t} \right)$ as ${\hat {\boldsymbol{S}}_{\mathrm{sag}}}\left( {{\boldsymbol{s}},\;t} \right) = \hat{\boldsymbol{I}}\left( { {\boldsymbol{v}}\left( {{\boldsymbol{x}},{\boldsymbol{y}},{\boldsymbol{z}}} \right){|_{x = {p_t}}},\;t} \right)$, with the slice location ${p_t}$ matching that of ${{\boldsymbol{S}}_{\mathrm{sag}}}$; ${\hat {\boldsymbol{S}}_{\mathrm{cor}}}\left( {{\boldsymbol{s}},\;t} \right) = \hat{\boldsymbol{I}}\left( { {\boldsymbol{v}}\left( {{\boldsymbol{x}},{\boldsymbol{y}},{\boldsymbol{z}}} \right){|_{{\boldsymbol{y}} = {\boldsymbol{p_t}}}},{\boldsymbol{t}}} \right)$, with slice location identical to that of ${{\boldsymbol{S}}_{\mathrm{cor}}}$.

In addition to the data consistency loss in equation (6), three regularization losses were implemented in stage II, including a total variation (TV) regularization, a normalization loss on the MBCs, and a smoothness loss on the DVFs.

The TV loss was adopted as an image-domain regularization to suppress high-frequency image noise/artifacts while preserving anatomical edges:

\begin{equation*}{\mathcal{L}_{\mathrm{TV}}} = \frac{1}{{{N_{\mathrm{voxel}}}}}\mathop \sum \limits_{i = 1}^{{N_{\mathrm{voxel}}}} \left| {\nabla {{\hat {\boldsymbol{I}}}_{\mathrm{ref}}}\left( {{ {\boldsymbol{v}}_i}} \right))} \right|,\end{equation*}

where ∇ denotes the gradient operator, which was calculated using forward finite difference.

The normalization loss of MBCs was defined as:

\begin{equation*}{\mathcal{L}_{\mathrm{MBC}}} = \frac{1}{{3L}}\mathop \sum \limits_{i = 1}^L \mathop \sum \limits_{j = x,y,z} {\left| {{{\widehat {\boldsymbol{e}}}_{i,j}}{{\left( {\boldsymbol{v}} \right)}^2} - 1} \right|^2}\end{equation*}

where the subscript i and j=x,y,z represent the MBC level index, and the Cartesian components index, respectively.

The smooth loss calculated the mean deformation energy of the DVF via:

\begin{equation*}{\mathcal{L}_{\mathrm{smooth}}} = \frac{1}{{{N_{\mathrm{voxel}}}}}\mathop \sum \limits_{i = 1}^{{N_{\mathrm{voxel}}}} \mathop \sum \limits_{j = x,y,z} \left( {{{\left( {\frac{{\partial {{\hat d}_j}\left( {{ {\boldsymbol{v}}_i}} \right)}}{{\partial x}}} \right)}^2} + {{\left( {\frac{{\partial {{\hat d}_j}\left( {{ {\boldsymbol{v}}_i}} \right)}}{{\partial y}}} \right)}^2} + {{\left( {\frac{{\partial {{\hat d}_j}\left( {{ {\boldsymbol{v}}_i}} \right)}}{{\partial z}}} \right)}^2}} \right).\end{equation*}

The total loss function in stage II was a weighted sum of the four losses:

\begin{equation*}{\mathcal{L}_{\mathrm{total}}} = \mathcal{L}_{\mathrm{sim}}^{slc} + {{{\lambda }}_{\mathrm{TV}}}{\mathcal{L}_{\mathrm{TV}}} + {{{\lambda }}_{\mathrm{MBC}}}{\mathcal{L}_{\mathrm{MBC}}} + {{{\lambda }}_{\mathrm{sh}}}{\mathcal{L}_{\mathrm{smooth}}},\end{equation*}

where ${\boldsymbol{\lambda }}$ denote the weighting hyperparameters.

Given the one-shot training setting, the learned motion encoder may be less robust to respiratory motion patterns underrepresented during training. To address this issue, a motion score augmentation strategy (Shao *et al* 2025a) has been developed for S2V-DREME. Augmented MBC scores were generated by randomly perturbing the learned MBC scores as

\begin{equation*}{\boldsymbol{\hat w}}^{^{\prime} _{i,j}}\left( {\mathrm{t}} \right) = {r_1}\left( t \right) \times {r_2}\left( {i,j} \right) \times {{\boldsymbol{\hat w}}_{i,j}}\left( t \right),\end{equation*}

where ${r_1} \in \left[ {0.4,{\text{ }}1.5} \right]$ and ${r_2} \in \left[ {0.8,{\text{ }}1.2} \right]$ were uniformly distributed random variables. ${r_1}\left( t \right)$ provided an overall motion-amplitude perturbation for each slice pair, ${r_2}\left( {i,j} \right)$ introduced level- and direction-specific variations across MBC scores. The augmentation loss was defined as:

\begin{equation*}{\mathcal{L}_{\mathrm{Aug}}} = \frac{1}{{3L}}\mathop \sum \limits_{i = 1}^L \mathop \sum \limits_{j = x,y,z} {\left| {{\boldsymbol{\hat w}}_{i,j}^{\prime\prime}\left( {\mathrm{t}} \right) - {\boldsymbol{\hat w}}_{i,j}^{\prime}\left( {\mathrm{t}} \right)} \right|^2},\end{equation*}

where $\widehat {\mathbf{w}}_{i,j}^{^{\prime\prime}}\left( {\mathrm{t}} \right)$ denotes the MBC score predicted by the motion encoder.

In the augmentation training stage following the main S2V-DREME training, the learned reference volume and MBCs were fixed, while the FiLM-based motion encoder was further optimized. Augmented DVFs were computed from the perturbed MBC scores and learned MBCs, and were applied to the reference volume to generate motion-augmented dynamic volumes. Augmented orthogonal slice pairs were then extracted from these volumes and fed into the encoder to predict ${\boldsymbol{\hat w}}_{i,j}^{^{\prime\prime}}\left( {\mathrm{t}} \right)$. The encoder was optimized using ${\mathcal{L}_{\mathrm{Aug}}}$ to match the augmented MBC scores ${\boldsymbol{\hat w}}_{i,j}^{\prime}\left( {\mathrm{t}} \right)$.

In the above sections, the described data input settings focus on the orthogona-view acquisition protocol. They can be conveniently adapted to other step-and-shoot acquisition scenarios (single-view single-slice or single-view multi-slice acquisitions), which were also investigated in this study.

### 2.5. Evaluation datasets and schemes

S2V-DREME was evaluated under three experimental settings: a digital phantom simulation, a physical phantom measurement study, and a human subject measurement study. The digital phantom experiments were performed using the extended cardiac torso (XCAT) phantom (Shao *et al* 2025b), which provided ground-truth images and motion trajectories for method development, hyperparameter analysis, and ablation study. The physical phantom data were acquired on a 1.5 T clinical MRI system (Ingenia Ambition, Philips N.V., Netherlands), for which ‘ground-truth’ motion was available, enabling quantitative evaluation. Following validation on the phantom datasets, S2V-DREME was further tested on four healthy volunteers with the same 1.5 T scanner to qualitatively assess its performance. The experimental setup and preprocessing procedures for each dataset are detailed below.

#### 2.5.1. XCAT simulation study

Five respiratory motion scenarios with irregularities in breathing frequency, amplitude, and baseline drift were simulated by XCAT to evaluate the performance of S2V-DREME, as summarized in table 1. For each motion trajectory, 1500 XCAT volumes were generated at a sampling rate of 0.35 volumes/s. Each volume had a spatial size of 150 (SI direction) × 180 × 180 voxels and a voxel resolution of 1.5 × 1.5 × 1.5 mm^3^. A spherical tumor with a diameter of 3 cm was inserted into the liver to enable quantitative motion tracking.

**Table 1.** Motion characteristics of the five motion scenarios used in the simulation study.

Motion scenarios	Motion characteristics
Motion 1	Amplitude variations
Motion 2	Baseline drifts
Motion 3	Amplitude variations and baseline drifts
Motion 4	Respiratory period/amplitude variations
Motion 5	Respiratory period/amplitude variations and baseline drifts

For each motion scenario, ‘one-shot’ training dataset was constructed by sampling orthogonal 2D slice pairs from the dynamic volumes along the sagittal and coronal views. To simulate realistic slice thickness, slice averaging was applied over three adjacent slices, resulting in an effective slice thickness of 4.5 mm. A total of P=180/3=60 slice locations were yielded for each view. At each location, T=20 consecutive orthogonal slice pairs were sampled with a temporal resolution of 0.35 s per pair. For each motion trajectory, a 420 s segment corresponding to 1200 orthogonal slice pairs was used for training, while the remaining 300 orthogonal slice pairs, acquired at fixed central slice locations, were reserved for real-time inference.

The original XCAT volumes were used as references to assess reconstruction accuracy. Image quality was evaluated using intensity-based metrics, including the structural similarity index (SSIM) and relative error (RE). The structural similarity between the reconstructed volume ${\hat {\boldsymbol{I}}_{\mathrm{rec}}}$ and the reference (GT) volume ${{\boldsymbol{I}}_{\mathrm{GT}}}$ was quantified using SSIM (Wang *et al* 2004), which was defined as follows:

\begin{equation*} \mathrm{SSIM} = \frac{\left(2\hat{\mu}_{\mathrm{rec}}\mu_{\mathrm{GT}} + {\mathrm{c}}_1\right)\left(2\mathrm{cov}\left(\hat{\boldsymbol{I}}_{\mathrm{rec}}, \boldsymbol{I}_{\mathrm{GT}}\right) + {\mathrm{c}}_2\right)}{\left(\hat{\mu}_{\mathrm{rec}}^2 + \mu_{\mathrm{GT}}^2 + {\mathrm{c}}_1\right)\left(\hat{\sigma}_{\mathrm{rec}}^2 + \sigma_{\mathrm{GT}}^2 + {\mathrm{c}}_2\right)} \end{equation*}

where ${{\mu }}$ denotes the means, ${{\sigma }}$ denotes the variances, ${\mathrm{cov}}\left( \cdot \right)$ is the covariance, and ${{\mathrm{c}}_1},{\text{ }}{{\mathrm{c}}_2}$ are constants to avoid numerical instability.

The RE was quantified by measuring the intensity difference between the reconstructed volume ${\hat {\boldsymbol{I}}_{\mathrm{rec}}}$ and the reference volume ${{\boldsymbol{I}}_{\mathrm{GT}}}$:

\begin{equation*}{\mathrm{RE}} = \sqrt {\frac{{\mathop \sum \nolimits_{{{\mathrm{N}}_{{\mathrm{voxel}}}}} {{\left( {{{\hat {\boldsymbol{I}}}_{\mathrm{rec}}} - {{\boldsymbol{I}}_{\mathrm{GT}}}} \right)}^2}}}{{\mathop \sum \nolimits_{{{\mathrm{N}}_{{\mathrm{voxel}}}}} {\boldsymbol{I}}_{\mathrm{GT}}^2}}} \times 100\% {\mathrm{.}}.\end{equation*}

Motion tracking accuracy was evaluated using the liver tumor as the target, and quantified through contour-based metrics, including the 95th percentile Hausdorff distance (HD95) (Taha and Hanbury 2015), the Dice similarity coefficient (DSC) (Dice 1945), and the center-of-mass error (COME) (Zhang *et al* 2015).

Let ${{\boldsymbol{\hat M}}_{{\mathrm{rec}}}} \in \left\{ {0,1} \right\}$ and ${{\mathbf{M}}_{{\mathrm{GT}}}} \in \left\{ {0,1} \right\}$ represent the reconstructed and ‘GT’ liver tumor masks, respectively. The HD95 is calculated as:

\begin{equation*}{\mathrm{HD}}95 = \max \left\{ {{{\mathrm{P}}_{95}}{\mathrm{d}}\left( {{{\hat{\mathrm{m}}}_{{\mathrm{rec}}}},{{\mathbf{M}}_{{\mathrm{GT}}}}} \right),{{\mathrm{P}}_{95}}{\mathrm{d}}\left( {{{\mathrm{m}}_{{\mathrm{GT}}}},{{{\boldsymbol{\hat M}}}_{{\mathrm{rec}}}}} \right)} \right\},\end{equation*}

where ${{\mathrm{P}}_{95}}$ is the 95-percentile operator, and ${\mathrm{d}}\left( {{{\hat{\text{ m}}}_{{\mathrm{rec}}}},{{\mathbf{M}}_{{\mathrm{GT}}}}} \right) = \mathop {\min }\limits_{{{\hat{\mathrm{m}}}_{{\mathrm{rec}}}} \in {{{\boldsymbol{\hat M}}}_{{\mathrm{rec}}}}} {\left\| {{{\hat{\text{ m}}}_{{\mathrm{rec}}}} - {{\mathbf{M}}_{{\mathrm{GT}}}}} \right\|_2}$ is the distance from a reconstructed voxel ${\hat{\mathrm{m}}_{{\mathrm{rec}}}}$ to the ‘ground-truth’ mask ${\text{ }}{{\mathbf{M}}_{{\mathrm{GT}}}}$. Similarly, ${\mathrm{d}}\left( {{{\mathrm{m}}_{{\mathrm{GT}}}},{{{\boldsymbol{\hat M}}}_{{\mathrm{rec}}}}} \right)$ is the distance from a ‘ground-truth’ contour voxel ${{\mathrm{m}}_{\mathrm{T}}}$ to the reconstructed mask ${{\boldsymbol{\hat M}}_{{\mathrm{rec}}}}$.

The Dice measures the similarity between ${{\boldsymbol{\hat M}}_{{\mathrm{rec}}}}$ and ${{\mathbf{M}}_{{\mathrm{GT}}}}$ by evaluating their spatial overlap, as follows:

\begin{equation*}{\mathrm{Dice}} = \frac{{2\left| {{{{\boldsymbol{\hat M}}}_{\mathrm{rec}}} \cap {{\mathbf{M}}_{{\mathrm{GT}}}}} \right|}}{{\left| {{{{\boldsymbol{\hat M}}}_{{\mathrm{rec}}}}} \right| + \left| {{{\mathbf{M}}_{{\mathrm{GT}}}}} \right|}},\end{equation*}

where Dice = 0 means that ${{\boldsymbol{\hat M}}_{{\mathrm{rec}}}}$ and ${{\mathbf{M}}_{{\mathrm{GT}}}}$ have no overlap, and Dice = 1 indicates that ${{\boldsymbol{\hat M}}_{{\mathrm{rec}}}}$ and ${{\mathbf{M}}_{{\mathrm{GT}}}}$ are identical.

The COME measures the center-of-mass distance between ${{\boldsymbol{\hat M}}_{{\mathrm{rec}}}}$ and ${{\mathbf{M}}_{{\mathrm{GT}}}}$, defined as:

\begin{equation*}{\mathrm{COME}} = {\hat{\mathrm{C}}_{{\mathrm{rec}}}} - {{\mathrm{C}}_{{\mathrm{GT}}}}_2,\end{equation*}

where ${\hat{\text{ C}}_{{\mathrm{rec}}}}$ and ${{\mathrm{C}}_{{\mathrm{GT}}}}$ denote the centers-of-mass of ${{\boldsymbol{\hat M}}_{{\mathrm{rec}}}}$ and ${{\mathbf{M}}_{{\mathrm{GT}}}}$, respectively.

#### 2.5.2. Physical phantom study

To evaluate the performance of S2V-DREME under realistic acquisition conditions, a physical phantom study was conducted using a CIRS-008Z MRgRT motion management QA phantom (Burton *et al* 2025), consisting of an MR-compatible body and a cylindrical insert containing a tumor target. The insert was driven along the SI direction by piezoelectric motors using predefined motion waveforms. Three motion trajectories with varying amplitudes and frequencies were applied to generate distinct respiratory motion profiles. Using an in-house 1.5 T Philips clinical MR scanner, orthogonally interleaved 2D cine pairs in the sagittal and coronal views were acquired at 10 consecutive slice locations. The acquisition used a single-shot, turbo-spin echo (SS-TSE) sequence for ultra-fast, T2-weighted imaging (Wang *et al* 2007). The corresponding parameters are: TR = 250 ms, TE = 20 ms, and Flip Angle = 90°. Due to the lack of a ready-to-use, continuous ‘step-and-shoot’ protocol (figure 1), we manually concatenated orthogonal cine scans with stepping locations to traverse the 3D volume. Due to the concatenation nature, a short temporal gap (∼4–5 s) exists between acquisitions of consecutive slice locations. Previous studies have shown that automatic 2D slice stepping can be achieved to eliminate the time gap (Liu 2016). The original images had an in-plane resolution of 0.762 × 0.762 mm^2^ with a matrix size of 528 × 528 and were subsequently resampled to 2.5 × 2.5 mm^2^ before reconstruction. The slice thickness was 6 mm. At each slice location, 20 orthogonal slice pairs were collected with a temporal resolution of ∼0.5 s per pair. Motion tracking accuracy was quantitatively evaluated using the COME, which measured the similarity between the estimated SI motion trajectory and the ground-truth trajectory.

#### 2.5.3. Human subject study

S2V-DREME was further evaluated using *in vivo* human data acquired from four healthy volunteers to assess its robustness and feasibility under realistic imaging/clinical conditions. All human data were acquired on the same 1.5 T Philips clinical MR scanner used in physical phantom experiments. For each subject, orthogonal 2D slice pairs were continuously acquired along the sagittal and coronal planes across 25–28 slice locations, covering the thoracic-abdominal region. The same orthogonal-view SS-TSE imaging sequence and parameters as those used in the physical phantom study were applied in the human study. At each location, 20 consecutive slice pairs were acquired with a temporal resolution of approximately 0.5 s per pair. The original in-plane resolution (0.903 × 0.903 mm^2^, matrix size 288 × 288) was resampled to 2.0 × 2.0 mm^2^ for reconstruction, with a slice thickness of 6 mm.

### 2.6. Implementation details

As mentioned in section 2.4, the progressive training scheme first warm-started the spatial INR and the motion model separately, followed by joint optimization of the entire framework to improve accuracy and ensure consistency between the solved anatomy/motion and the 2D slice data. After initializing the spatial INR for ${N_a} = 1000$ epochs, Stage II performed a multi-resolution initialization of the motion model. The training process started from the lowest spatial resolution (L=1) of the MBCs and their corresponding scores and progressively incorporated higher spatial resolutions. For phantom studies, we used two resolution levels L= 2 and for human studies, three resolution levels were used to describe more complex motion (L= 3). Each resolution level was trained for 800 epochs. During the first ${\text{ }}{N_{b1,{\text{ }}L}} = 200$ epochs at each level, the spatial INR and the hash encoder were temporarily frozen, followed by joint optimization for ${N_{b2,{\text{ }}L}} = 600$ epochs. This procedure was repeated for all MBC levels.

Stage I training was conducted using the $\mathcal{L}_{\mathrm{sim}}^{ref}$ loss with a learning rate of $5 \times {10^{ - 4}}$. In Stage II, the model was optimized with the ${\mathcal{L}_{\mathrm{total}}}$ loss. The learning rates were set to 1$ \times {10^{ - 4}}$ for the spatial INR and 5$ \times {10^{ - 4}}$ for the motion model. The weighting coefficients were ${{{\lambda }}_{\mathrm{TV}}}$ = 2$ \times {10^{ - 6}}$, ${\text{ }}{{{\lambda }}_{\mathrm{MBC}}}$ = 1$ \times {10^{ - 2}}$, and ${\text{ }}{{{\lambda }}_{\mathrm{sh}}}$ = 1$ \times {10^{ - 3}}$, empirically determined by trial-and-error experiments. The Adam optimizer was employed for mini-batch training with a batch size of 32. The framework was implemented in PyTorch (v1.13), and all experiments were conducted on a single NVIDIA RTX 4090 GPU. The control-point spacing in the SINR-based MBC generator was set to (5, 3) for the 2 resolution-level XCAT and physical phantom studies, and to (7, 5, 3) for the three-resolution-level human subject study.

### 2.7. Ablation study, hyperparameter analysis, and acquisition sequence evaluation

To evaluate the effectiveness of the key components in the proposed S2V-DREME framework, we performed two groups of ablation experiments on the XCAT dataset. All methods were evaluated across five simulated motion scenarios, and quantitative performance was assessed using the mean SSIM, RE, DSC, HD95, and COME. The first group of ablation experiments investigated the impact of slice input strategies and motion encoder design. We compared the proposed S2V-DREME with four ablation variants:

(1) SagittalView, where only a single sagittal view was provided as input to the S2V-DREME framework (for both the motion encoder and loss computation);
(2) CoronalView, which used a single coronal view as input;
(3) TwoView, where two orthogonal slices were used without incorporating slice location information for the motion encoder;
(4) TwoV + PosIn, which further incorporated slice location as an additional input to the motion encoder, without FiLM-based modulation.

The second group of ablation experiments focused on the design of the MBC generator. Specifically, we compared the proposed SINR-based MBC generator with a baseline that generated MBCs using learnable B-spline interpolants (Tegunov and Cramer 2019) (referred to as BI-S2V-DREME) under different total training epoch settings on the XCAT dataset, in order to evaluate the impact of the MBC generation strategy on training efficiency. For comparison, different combinations of training epochs were evaluated with ${\text{ }}L = 2,$ ${N_a}{\text{ fixed at }}1000$, ${N_{b1}}$ fixed at 200, and ${N_{b2}}$ = {300, 600, 800, 1000, 1300}, corresponding to total epochs of {2000, 2600, 3000, 3400, 4000}, respectively.

The proposed S2V-DREME framework involved two key hyperparameters. The first was the number of 2D slice pairs acquired per position, which determines sampling sufficiency and influences both acquisition time and reconstruction accuracy. A systematic hyperparameter analysis was performed by setting the number of slices per position to {5, 10, 15, 20, 25, 30}, and evaluating the model on the XCAT dataset across five motion scenarios. The second hyperparameter was the control-point spacing in the SINR-based MBC generator. To investigate the effect of different control-point densities on model performance, three control-point spacing settings were evaluated: (7, 5), (5, 3), and (3, 2).

For the XCAT study, we compared S2V-DREME with a retrospective sorting-based 4D-MRI reconstruction method, in which 2D cine images were phase- or amplitude-sorted to generate 4D volumes (Abdelnour *et al* 2007, Liu *et al* 2014). Specifically, we sorted 20 consecutive sagittal slices acquired at each slice location into 10 respiratory phase/amplitude bins according to the known ‘ground-truth’ respiratory motion trajectory (we used the sagittal view here, as the cross-slice lateral motion is small). Here, we assume the real motion trajectory is known for 4D sorting, which presents an ideal scenario for sorting-based 4D-MRI reconstruction. For the same slice location, as the slices are over-sampled (20 slices for 10 bins), more than one slice can be assigned to a single phase/amplitude bin. In this case, the slice closest to the phase/amplitude bin center was selected. If no slice was available for a given bin (due to motion variability), the slice from the same location in the nearest adjacent bin was used as a substitute, with preference given to the preceding bin; if the preceding bin was unavailable (e.g. bin 1), the subsequent bin was used. With 10 slices assigned to 10 bins at each location, we concatenated the slices of the same bin along the slice direction to form a bin-specific volume, rendering a 10-phase 4D-MRI. Then, based on the tagged bin of each slice, we assigned a corresponding 4D-MRI volume with the matching bin to represent the volumetric anatomy at that instance. For these volumes, we evaluated the reconstruction accuracy using SSIM, RE, DSC, HD95, and COME, by comparing each volume with its corresponding ground-truth XCAT volumes associated with the sampled slices. Both the phase- and amplitude-sorting methods are retrospective reconstruction approaches that rely on complete data acquisition for 4D-MRI and are incapable of real-time image and motion estimation.

The step-and-shoot acquisition protocol alternated between two orthogonal slice orientations in the SS-TSE sequence, which could introduce dark-band artifacts and affect subsequent motion estimation. To mitigate this issue, balanced fast field echo (BFFE) was investigated as an alternative acquisition sequence in this study (Tyagi *et al* 2021, Jassar *et al* 2023). Compared with SS-TSE, BFFE is a balanced steady-state free precession sequence that provides mixed T2/T1-weighted contrast and maintains more stable steady-state magnetization. In SS-TSE, repeated alternation between sagittal and coronal slice acquisitions may disturb magnetization recovery and the refocusing process, leading to signal inconsistencies that appear as dark-band artifacts. In comparison, the steady-state signal formation in BFFE is less dependent on the long echo-train refocusing process used in SS-TSE, potentially making BFFE less susceptible to orientation-switching-induced signal variations. Additional experiments were conducted on the physical phantom under motion scenario P3 and on one volunteer to evaluate BFFE-based orthogonal-view acquisition. For the phantom study, the original images had an in-plane resolution of 0.998 × 0.998 mm^2^ with a matrix size of 400 × 400 and were resampled to 2.5 × 2.5 mm^2^ before reconstruction, with a slice thickness of 5 mm. For the volunteer study, the original images had the same in-plane resolution and matrix size and were resampled to 2.0 × 2.0 mm^2^ before reconstruction, with a slice thickness of 4 mm. The temporal resolution was approximately 0.5 s per slice pair in both studies.

Single-view SS-TSE acquisition using the sagittal orientation was also evaluated on the same physical phantom under motion scenario P3 and the same volunteer as another strategy to mitigate dark-band artifacts. Both acquisitions comprised 20 consecutive sagittal frames at each slice location, with a temporal resolution of approximately 250 ms per frame. The slice thickness was 5 mm for the phantom and 4 mm for the volunteer, and all other acquisition settings followed those of the orthogonal-view SS-TSE acquisition. The volunteer study further included a single-view multi-slice BFFE acquisition comprising 20 consecutive three-slice sets at three sagittal locations, with an acquisition time of approximately 450 ms per set and a slice thickness of 5 mm. In this acquisition, the three parallel BFFE slices were acquired with fixed inter-slice gaps at each time frame, while the slice positions were progressively shifted across time to sample the gaps and cover the full volume. All other acquisition and reconstruction settings were consistent with those of the orthogonal-view BFFE acquisition. To account for potential intensity fluctuations, intensity normalization was performed both within and across slice positions for the orthogonal-view and single-view BFFE datasets.

In addition, we evaluated the effect of motion score augmentation (section 2.4) on the robustness of S2V-DREME to unseen motion variations using the XCAT dataset. S2V-DREME was trained separately on each of the five respiratory motion scenarios with and without motion score augmentation, and each trained model was tested on all five scenarios for real-time motion estimation.

## 3. Results

### 3.1. The XCAT study results

Table 2 summarizes the comparison with conventional 4D-MRI baseline methods (Sagittal-based Amplitude Sorting and Phase Sorting) in terms of image reconstruction quality and motion tracking accuracy on the XCAT dataset. The NA setting served as a reference baseline, in which no registration was applied, and the reference volume from S2V-DREME was directly compared with the ground-truth XCAT volumes. Compared with the NA baseline, the other methods improved reconstruction quality and motion tracking accuracy, yielding higher SSIM and Dice and lower HD95 and COME. However, the two sorting-based 4D-MRI methods relied on respiratory amplitude or phase information for sorting, which can be unreliable under irregular breathing patterns commonly encountered in clinical scenarios, leading to sorting errors and intra-phase-bin motion. In addition, the sorting-based methods assume regular motion and cannot resolve irregular motion patterns (especially for phase sorting). Compared with conventional 4D-MRI methods, the proposed S2V-DREME approach achieved substantial performance gains, yielding higher SSIM and Dice scores and lower RE, HD95, and COME values. Unlike the retrospective techniques, which were limited to nominal 4D volume reconstruction, S2V-DREME supported both dynamic reconstruction and real-time image and motion estimation to resolve both regular and irregular motion trajectories. The real-time estimation results were comparable to those achieved in dynamic reconstruction, demonstrating the model’s strong generalization capability and robustness to new, real-time motion patterns. Overall, these results demonstrated the effectiveness of the proposed method in improving image reconstruction quality and motion tracking accuracy in the presence of irregular respiratory motion.

**Table 2.** Quantitative comparison of image reconstruction quality and motion tracking performance between the proposed S2V-DREME method and 4D-MRI baseline methods (amplitude sorting and phase sorting) on the XCAT dataset. The 4D-MRI methods used the ground-truth breathing curve for amplitude or phase sorting, representing an ideal scenario. The NA setting served as a reference baseline without applying solved motion fields. The 4D-MRI reconstruction used the same training slices as S2V-DREME, while the real-time motion/image estimation capability of S2V-DREME was evaluated using a separate set of 300 slice pairs.

Metric	SSIM↑	RE↓	Dice↑	HD95(mm)↓	COME(mm)↓
NA	0.68 ± 0.03	0.32 ± 0.05	0.59 ± 0.07	8.72 ± 1.65	6.79 ± 0.82
4D-MRI: Sagittal amplitude sorting	0.78 ± 0.02	0.24 ± 0.03	0.78 ± 0.05	4.67 ± 1.49	2.23 ± 0.71
4D-MRI: Sagittal phase sorting	0.79 ± 0.03	0.23 ± 0.02	0.80 ± 0.04	4.52 ± 1.36	2.18 ± 0.53
S2V-DREME (dynamic recon)	0.83 ± 0.02	0.18 ± 0.02	0.92 ± 0.03	1.64 ± 0.78	0.98 ± 0.43
S2V-DREME (real-time estimation)	0.83 ± 0.02	0.17 ± 0.02	0.91 ± 0.02	1.55 ± 0.87	0.99 ± 0.73

**Figure 4.**
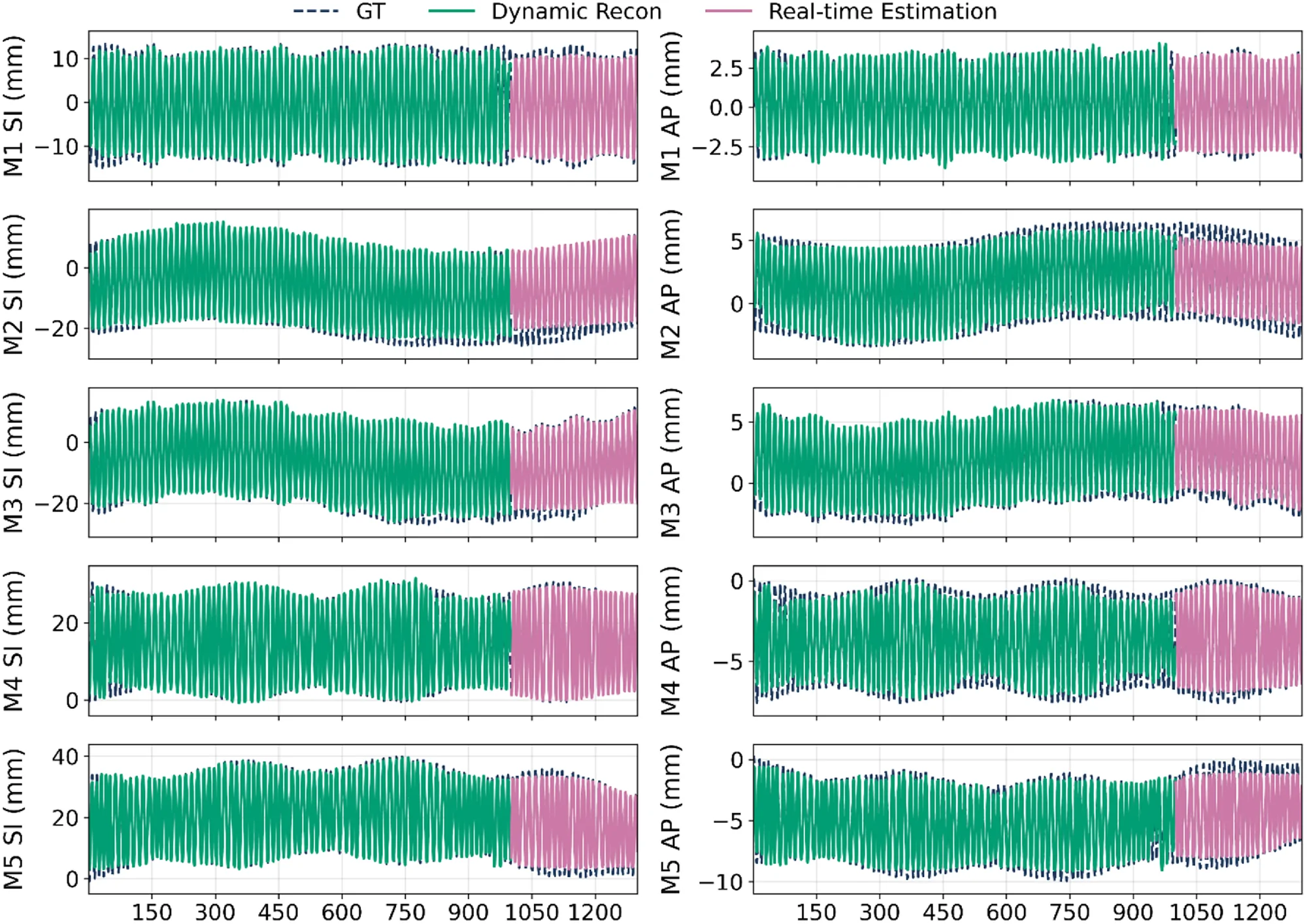
Comparison of S2V-DREME estimated and ground-truth tumor motion trajectories for XCAT scenarios M1–M5 in the SI (left) and AP (right) directions.

Figure 4 illustrates the motion trajectories along the SI and AP directions for the five motion scenarios in the XCAT study. The trajectories solved by S2V-DREME (both for the dynamic reconstruction and real-time estimation stages) were compared with the ground-truth trajectories. For all motion scenarios, the solved trajectories closely matched the GT in both directions, accurately capturing the motion amplitude, phase, and baseline variations. The real-time stage exhibited similar trajectory profile accuracy to those obtained in the dynamic reconstruction stage, despite being estimated from orthogonal slice measurements not included in training.

**Figure 5.**
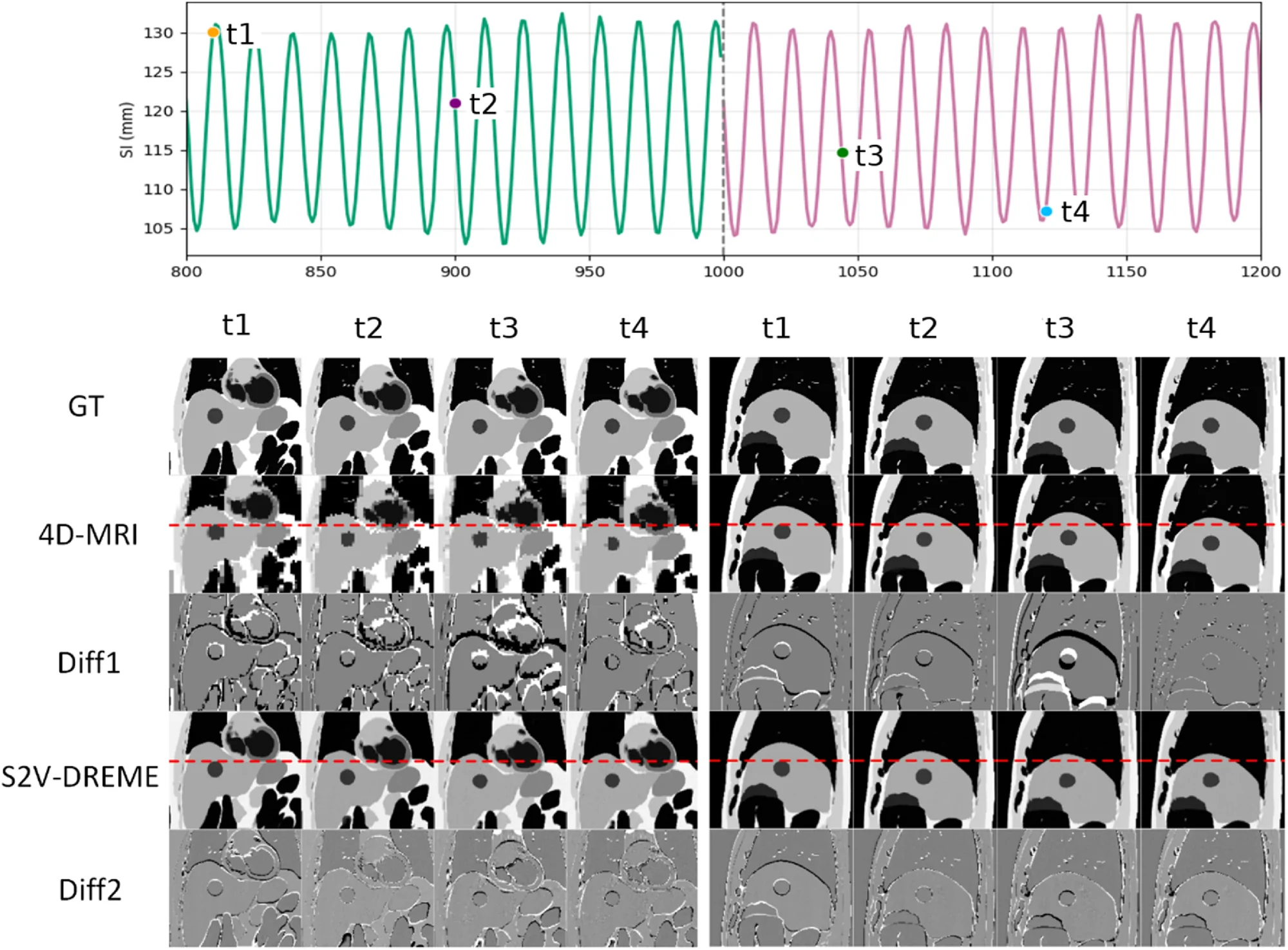
MR images generated by dynamic reconstruction and real-time estimation for XCAT (separated by the dashed line). The top row shows the estimated SI center-of-mass trajectory (M1). Remaining rows display ground truth (GT), 4D-MRI baseline (4D-MRI), their difference (Diff1), S2V-DREME results, and differences from GT (Diff2) in coronal (left) and sagittal (right) views.

Figure 5 presents motion-resolved MRI images from the XCAT study, with the dynamic reconstruction and real-time estimation stages separated by a dashed line. The top row shows the estimated SI center-of-mass trajectory, where four time points (*t*1–*t*4) were selected for visual comparison. The remaining rows displayed the corresponding GT, the conventional 4D-MRI baseline reconstructed using phase sorting, their difference maps (Diff1), the proposed S2V-DREME results, and the corresponding difference maps relative to the GT (Diff2), shown in both coronal and sagittal views. As illustrated in figure 5, the phase-sorted 4D-MRI baseline exhibited noticeable residual motion artifacts and anatomical inconsistencies, particularly around organ boundaries. These discrepancies were clearly visible in the corresponding difference maps, which reflected imperfect motion compensation due to phase-sorting inaccuracies. In contrast, the proposed S2V-DREME framework produced both dynamic reconstruction and real-time images that closely matched the GT across all selected time points. The difference maps of S2V-DREME showed substantially reduced residual errors, indicating improved structural consistency and motion tracking accuracy.

### 3.2. The phantom study results

**Figure 6.**
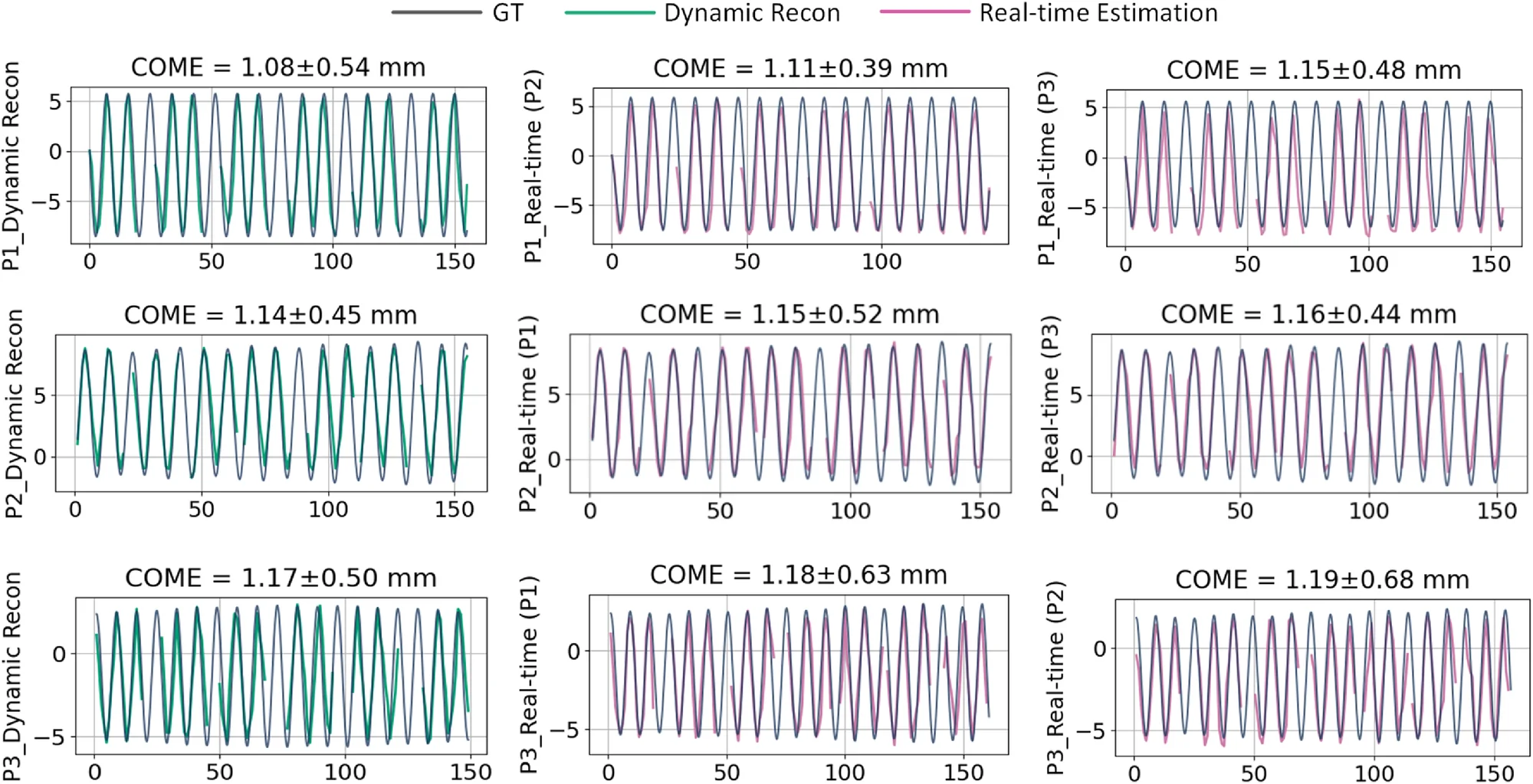
Comparison of solved and ground-truth motion trajectories for dynamic reconstruction and real-time motion/image estimation across three physical phantom measurements (P1–P3). ‘P1_dynamic recon’ denotes training and evaluation on the same phantom (P1), ‘P1_real-time (P2)’ denotes cross-measurement evaluation (training on P2 and evaluation on P1). Interruptions in the solved curves are due to changes in MR acquisition slice locations (stepping).

Figure 6 presents the solved motion trajectories and the corresponding ground-truth curves for three physical phantom measurements (P1–P3), under both intra-measurement dynamic reconstruction and cross-measurement real-time evaluation settings. Specifically, we used the acquired data from each measurement scenario for S2V-DREME training (intra-measurement dynamic reconstruction) and then applied the resulting model to directly infer real-time motion/images from the other two measurements (cross-measurement real-time motion/image estimation). The discontinuities observed in predicted curves were caused by the stepping of the MR slice location (section 2.5.2) during scanning. In the dynamic reconstruction scenario (training and evaluation on the same measurement), the predicted trajectories closely matched the GT trajectories, yielding COME values of 1.08 ± 0.54 mm (P1), 1.14 ± 0.45 mm (P2), and 1.17 ± 0.50 mm (P3), accurately capturing the programmed motion. For cross-measurement real-time estimation, where the model was trained on one measurement and evaluated on another, the estimated trajectories remained consistent with the GT. For P1 testing, the COME values were 1.11 ± 0.39 mm (trained on P2) and 1.15 ± 0.48 mm (trained on P3). For P2, the errors were 1.15 ± 0.52 mm (trained on P1) and 1.16 ± 0.44 mm (trained on P3). For P3, the COME values were 1.18 ± 0.63 mm (trained on P1) and 1.19 ± 0.68 mm (trained on P2). Although a modest increase in error was observed compared with the intra-measurement reconstruction setting, all COME values remained below 1.2 mm. Given that the real-time experiments were conducted under cross-measurement conditions, where the model was trained and evaluated on different motion patterns, the consistently low errors across all combinations demonstrated the model’s generalizability in the physical phantom experiments.

**Figure 7.**
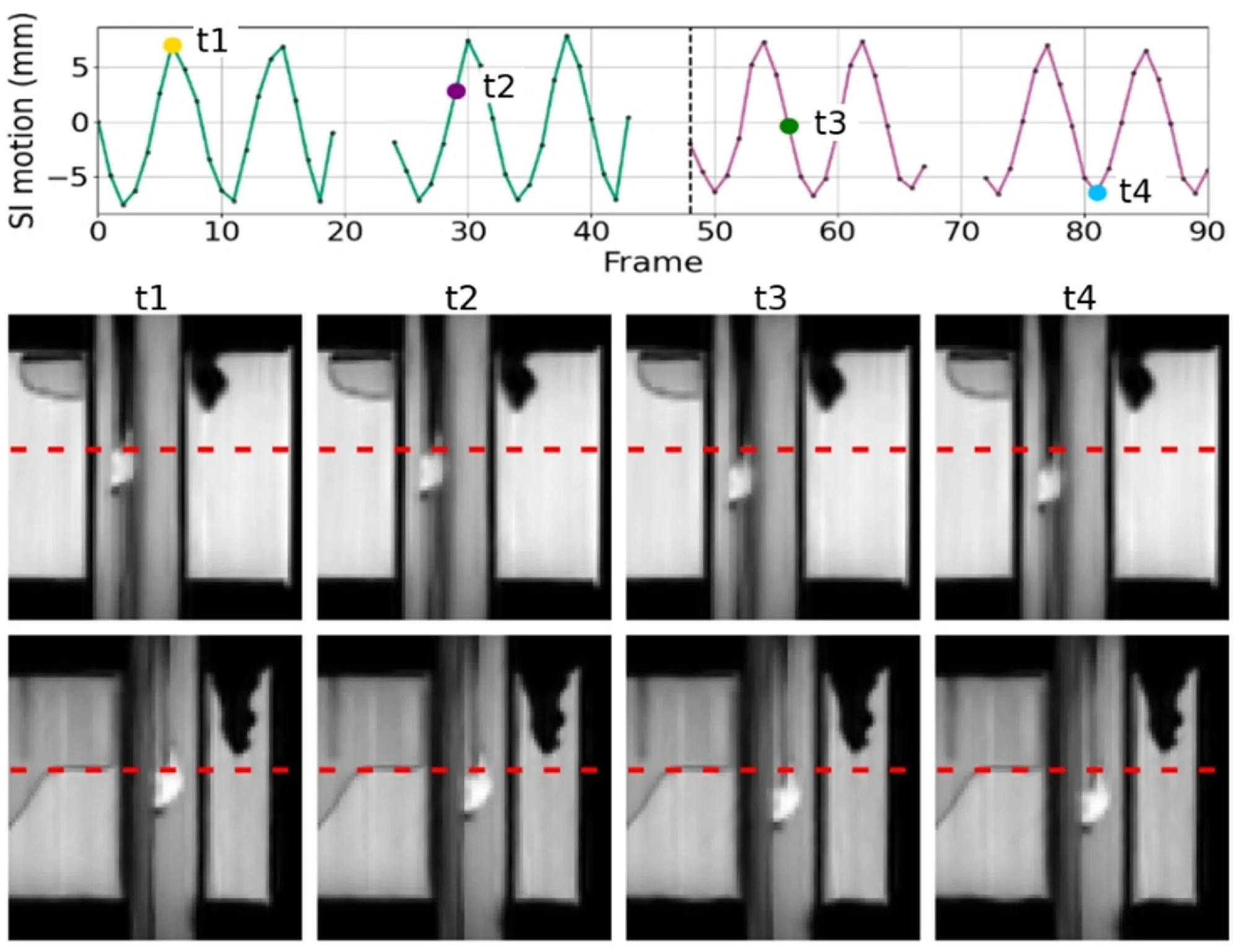
Time-resolved dynamic/real-time MR images of the phantom measurement studies. The first row presents the solved tumor center-of-mass trajectory along the SI direction. The labeled dots (*t*1–*t*4) indicate the selected time points for image visualization, and the vertical dashed line marks the separation between the intra-measurement dynamic reconstruction (training and evaluation on P1) and cross-measurement real-time evaluation (training on P2 and evaluation on P1). The subsequent rows show the reconstructed MR images in the sagittal and coronal views. The first two columns show the intra-measurement dynamic reconstruction, and the last two columns show the cross-measurement real-time evaluation.

Figure 7 illustrates example time-resolved MR images reconstructed from the phantom measurement study. The first row shows the estimated tumor center-of-mass trajectory along the SI direction, where four representative time points (*t*1–*t*4) were selected to sample different motion states. The reconstructed MR images at these time points were presented in the sagittal and coronal views for both intra-measurement dynamic reconstruction (training and evaluation on P1) and cross-measurement real-time evaluation (training on P2 and evaluation on P1). As observed, both tasks captured the varying motion with consistent motion trajectories, although some residual dark-band artifacts remained visible in the reconstructions.

### 3.3. The human study results

**Figure 8.**
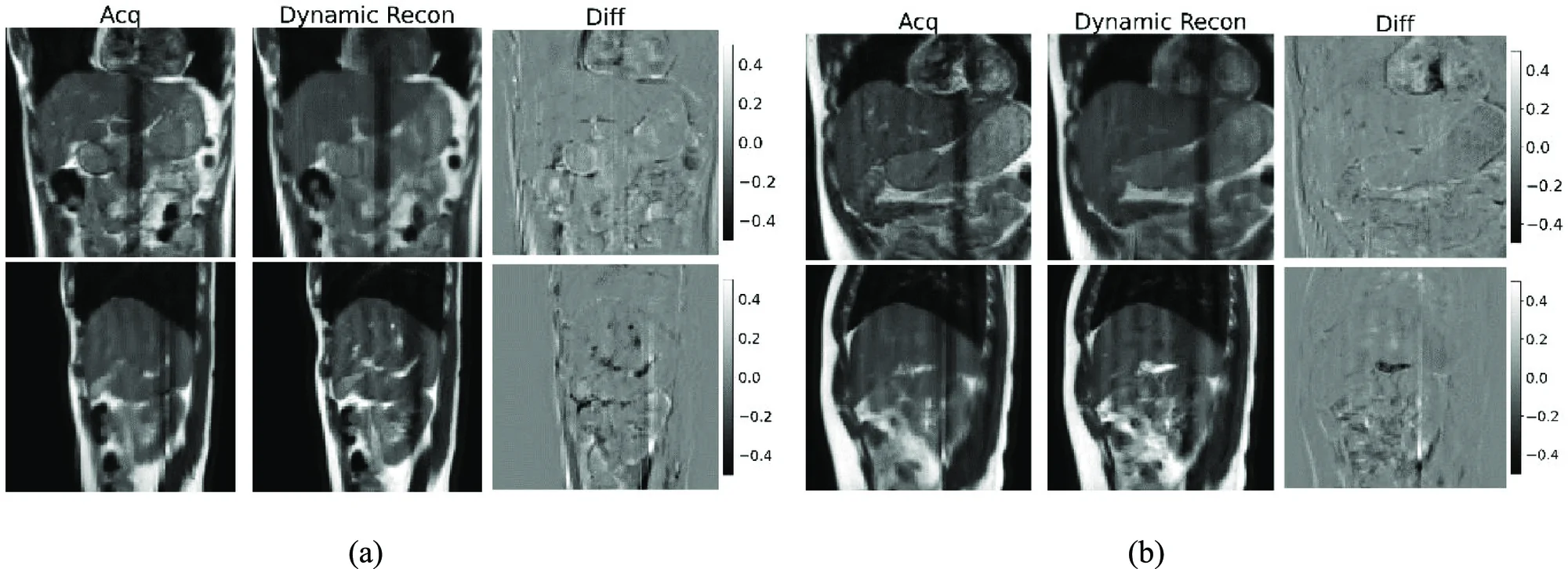
Visual comparison of acquired slices (Acq), extracted slices from reconstructed dynamic images (dynamic recon), and their corresponding difference maps (Diff) from two volunteers (a) and (b). The top row shows the coronal view, and the bottom row shows the sagittal view.

Figure 8 presents visual comparisons between the training slices (Acq) and the corresponding slices extracted from reconstructed dynamic 3D MRIs (dynamic recon) for two volunteers. The stripe-like dark-band artifacts observed in the images were associated with the orthogonal interleaved cine acquisition scheme. In SS-TSE imaging, switching between orthogonal slice orientations perturbs the magnetization steady state and slice history, leading to spatially varying T2 decay and saturation effects that manifest as dark band artifacts. Overall, the reconstructed slices retained the major anatomical structures observed in the acquired slices. The difference maps showed residual errors mainly around tissue interfaces and regions with strong intensity transitions. Stripe-like dark-band artifacts remained visible in the reconstructed slices as they are inherent in the acquired slices.

**Figure 9.**
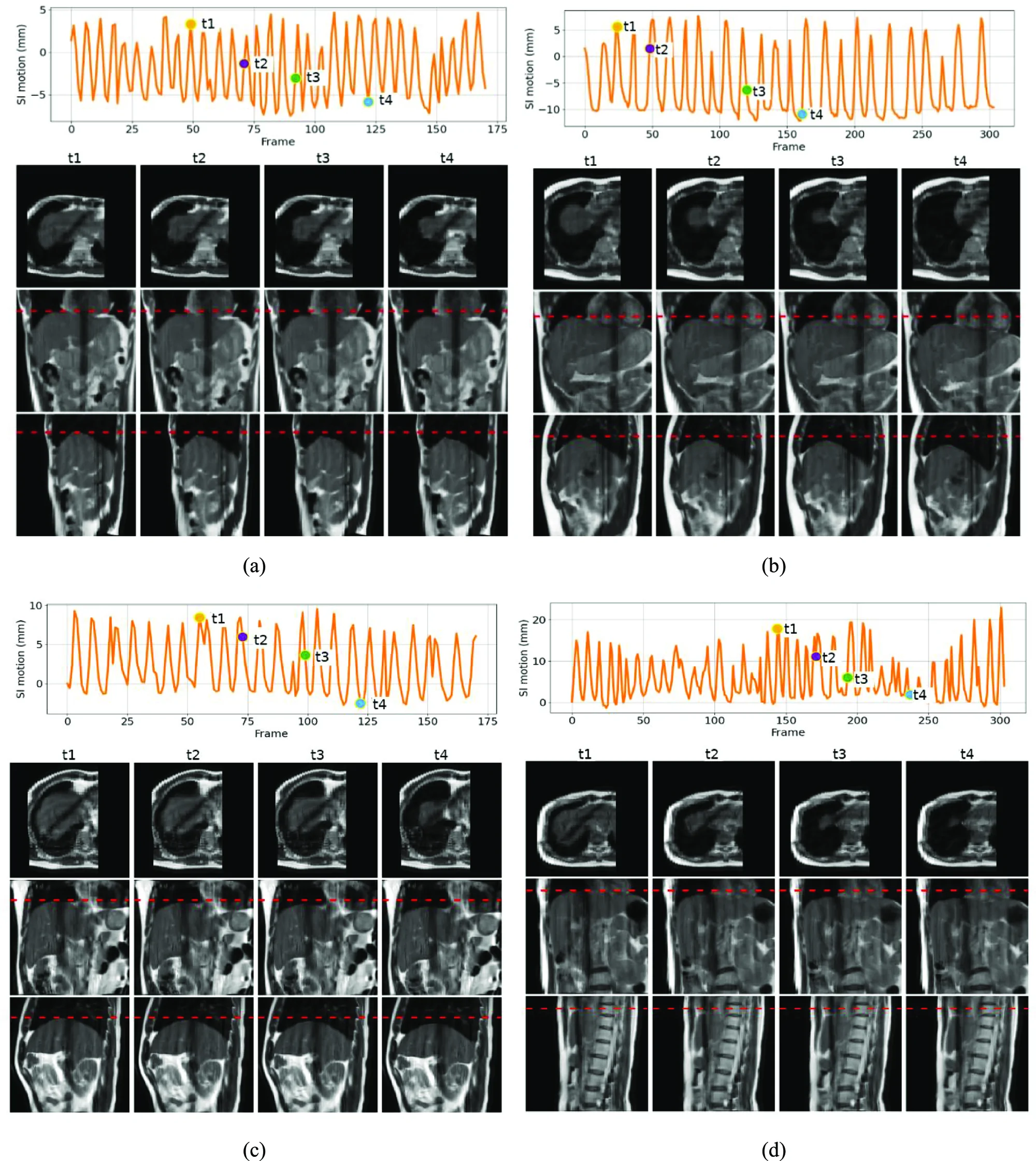
Time-resolved dynamic MR images of the four volunteers, (a)–(d). The first row shows the estimated liver center-of-mass trajectory along the SI direction. The subsequent rows display the reconstructed MR images in the axial, coronal, and sagittal views at four representative time points (*t*1–*t*4).

Figure 9 illustrates dynamic 3D MRIs reconstructed for the four volunteers (1–4), shown in subfigures (a)–(d). The first row displayed the estimated liver center-of-mass trajectory along the SI direction, with four representative time points (*t*1–*t*4) selected for visualization. The subsequent rows presented the reconstructed MR images at the corresponding time points in the axial, coronal, and sagittal views. The estimated liver motion trajectories exhibited respiratory motion with noticeable temporal and amplitude variations across subjects. The reconstructed images at different time points showed coherent liver displacement aligned with the estimated SI trajectories. Across all volunteers, anatomical structures remained spatially consistent in all three views, with generally preserved organ boundaries, although residual dark-band artifacts, which were propagated from the acquired slices, remained visible. Overall, these qualitative results suggest that the framework captured respiratory-related motion and enabled dynamic MR reconstruction in the evaluated volunteers.

### 3.4. Ablation study

**Figure 10.**
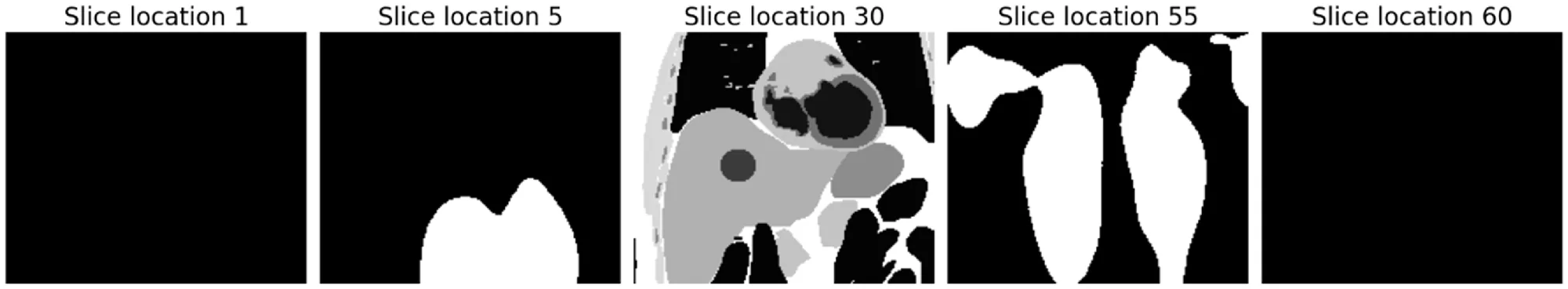
Visualization of coronal slices at five slice locations (1, 5, 30, 55, and 60) from an XCAT volume, with 60 slice locations in total.

Table 3 presents the quantitative results for different slice input strategies across five motion scenarios on the XCAT dataset. Slices at edge slice locations (1–5 and 55–60) contained little anatomical structure and had limited motion, leading to less reliable dynamic inference, as shown in figure 10. Therefore, two results were reported: Dynamic_AllLoc, which summarized reconstruction accuracy over all training slices (1200 slices), and Dynamic_NoEdgeLoc, which excluded edge slice locations when calculating the dynamic reconstruction accuracy. Only the results of Dynamic_NoEdgeLoc are presented in the subsequent sections. Note that both Dynamic_AllLoc and Dynamic_NoEdgeLoc are based on the same model trained on all 1200 slices, including the edge ones, and the difference is solely in how many slices are analyzed for reconstruction accuracy assessment. Real-time motion/image estimation results (table 3) based on the 300 leave-out slice pairs were also presented. For single-slice inputs, SagittalView generally achieved better performance than CoronalView across most evaluation metrics, yielding higher SSIM and DSC values and lower RE, HD95, and COME in both reconstruction and real-time tasks. The results were expected, as the sagittal view has access to the two directions with the most motion (SI and AP). When two orthogonal slices were used (TwoView), further improvements were observed across all metrics, indicating a consistent gain in accuracy compared with single-slice configurations. Incorporating slice location information as an additional input (TwoV + PosIn) led to further performance gains over TwoView for most metrics, particularly in terms of RE, HD95, and COME. Among all compared methods, the proposed S2V-DREME consistently achieved the best quantitative performance across all five motion scenarios, yielding the highest SSIM and DSC values while achieving the lowest RE, HD95, and COME for both reconstruction and real-time motion estimation.

**Table 3.** Ablation study of dynamic reconstruction and real-time motion estimation accuracy across five motion scenarios on the XCAT dataset. Metrics (SSIM, RE, DSC, HD95, and COME) were reported as mean ± standard deviation. Dynamic_AllLoc used all 1200 training slice pairs to calculate the dynamic reconstruction accuracy. Dynamic_NoEdgeLoc was based on 1000 slice pairs by excluding edge slice locations (1–5 and 55–60) of minimal anatomy/motion. For real-time motion estimation, evaluation was performed with 300 leave-out slice pairs acquired at the center of the liver tumor. The arrows point to the direction of improved accuracy.

Task	Metric	CoronalView	SagittalView	TwoView	TwoV + PosIn	S2V-DREME
Dynamic_AllLoc	SSIM↑	0.70 ± 0.14	0.75 ± 0.10	0.78 ± 0.06	0.79 ± 0.02	0.81 ± 0.05
	RE↓	0.28 ± 0.08	0.23 ± 0.09	0.20 ± 0.06	0.19 ± 0.04	0.18 ± 0.05
	DSC↑	0.77 ± 0.28	0.84 ± 0.21	0.85 ± 0.12	0.88 ± 0.06	0.89 ± 0.11
	HD95(mm)↓	3.64 ± 1.48	2.94 ± 1.48	2.85 ± 1.32	2.41 ± 0.65	2.52 ± 0.94
	COME(mm)↓	2.16 ± 0.78	2.11 ± 0.74	2.06 ± 0.71	1.87 ± 0.68	1.65 ± 0.83
Dynamic_NoEdgeLoc	SSIM↑	0.74 ± 0.07	0.77 ± 0.07	0.81 ± 0.02	0.82 ± 0.05	0.83 ± 0.02
	RE↓	0.24 ± 0.07	0.22 ± 0.07	0.19 ± 0.06	0.18 ± 0.05	0.18 ± 0.02
	DSC↑	0.80 ± 0.17	0.86 ± 0.17	0.87 ± 0.04	0.89 ± 0.12	0.92 ± 0.03
	HD95(mm)↓	2.86 ± 1.62	2.42 ± 1.14	2.31 ± 0.62	1.99 ± 1.52	1.64 ± 0.78
	COME(mm)↓	1.94 ± 0.50	1.75 ± 1.19	1.61 ± 0.92	1.14 ± 0.42	0.98 ± 0.43
Real-Time Motion/Image Estimation	SSIM↑	0.75 ± 0.07	0.77 ± 0.07	0.82 ± 0.02	0.82 ± 0.02	0.83 ± 0.02
	RE↓	0.23 ± 0.07	0.21 ± 0.07	0.19 ± 0.02	0.19 ± 0.04	0.17 ± 0.02
	DSC↑	0.82 ± 0.14	0.87 ± 0.17	0.88 ± 0.03	0.89 ± 0.09	0.91 ± 0.02
	HD95(mm)↓	2.51 ± 1.14	2.14 ± 1.48	2.07 ± 0.95	1.84 ± 1.02	1.55 ± 0.87
	COME(mm)↓	1.95 ± 1.27	1.54 ± 0.86	1.46 ± 0.78	1.24 ± 0.88	0.99 ± 0.73

**Figure 11.**
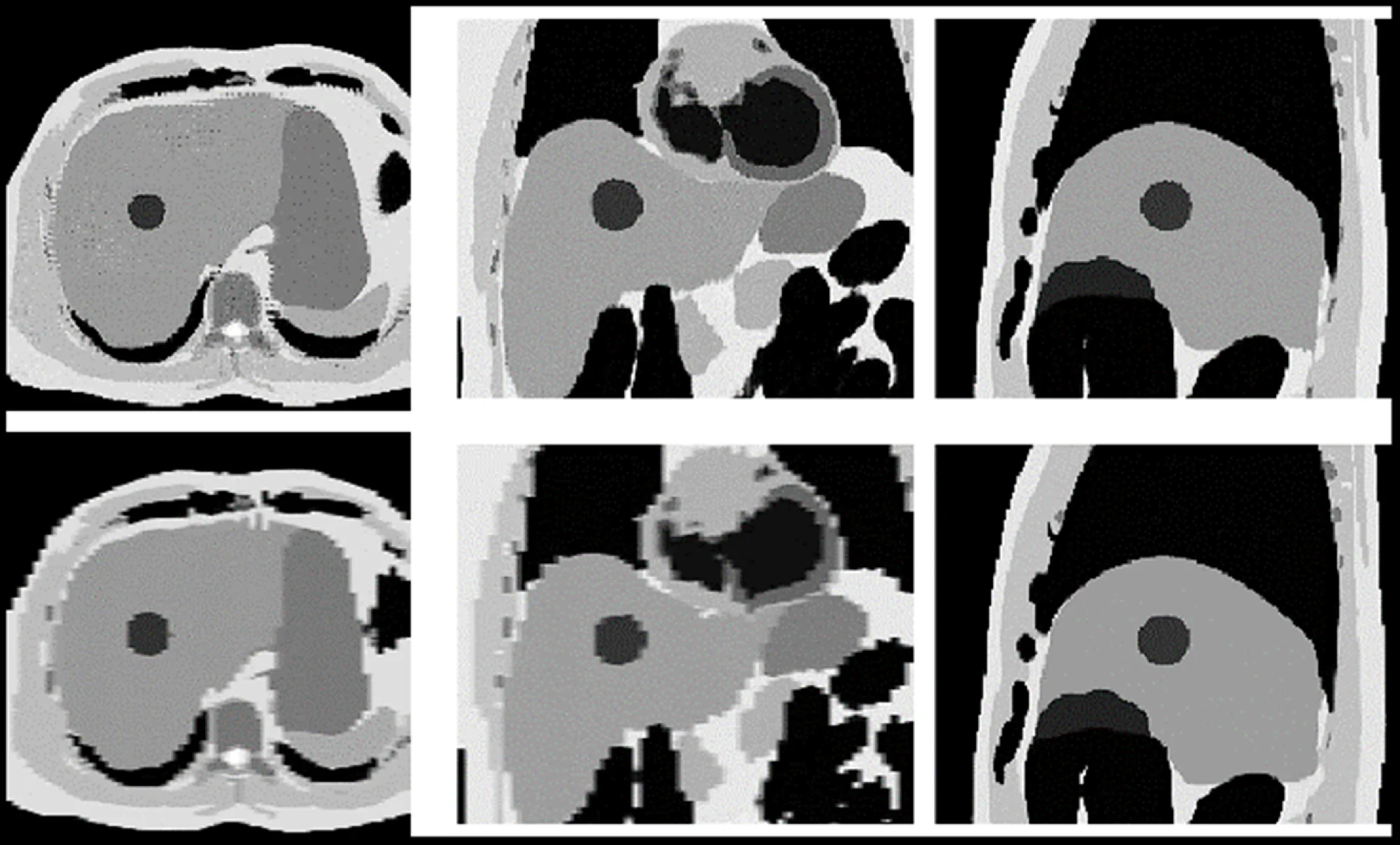
Comparison of reconstructed image resolution using orthogonal slice pairs (top) and sagittal-view-only slices (bottom) for S2V-DREME. The reconstructed reference anatomy is shown in axial, coronal, and sagittal views.

Figure 11 compares the reconstruction quality of S2V-DREME using orthogonal slice pairs and sagittal-view slices for training. When orthogonal slice pairs were used, the reconstructed reference anatomy exhibited high spatial resolution across the axial, coronal, and sagittal views. In contrast, reconstructions trained with sagittal-view-only slices preserved resolution mainly along the acquired plane, while resolution degradation occurred in the orthogonal (lateral) directions. This comparison suggested that incorporating orthogonal slice information helps to leverage the complementary, high in-plane resolutions from two views to improve the overall spatial resolution in the reconstructed volumes.

**Figure 12.**
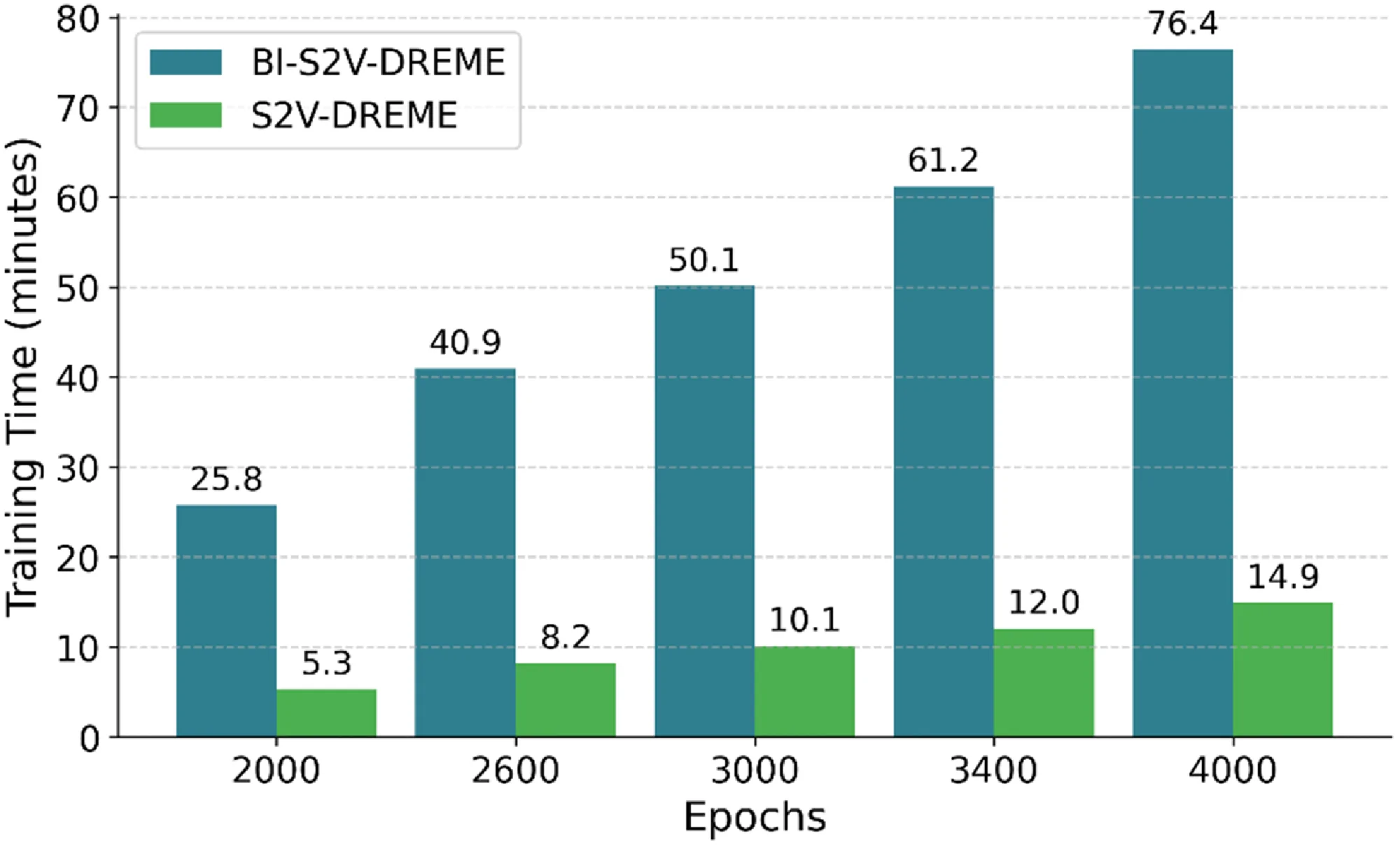
Training time comparison between B-spline-based (BI-S2V-DREME) and SINR-based S2V-DREME across different epoch settings on an NVIDIA RTX 4090 GPU.

Figure 12 illustrates the training time of BI-S2V-DREME and S2V-DREME on the XCAT dataset under different epoch settings. For both methods, the training time increased with the number of epochs. Across all evaluated epoch settings, SINR-based S2V-DREME consistently required substantially less training time than BI-S2V-DREME. Under a training configuration of 2600 epochs on an RTX 4090 GPU, S2V-DREME required ∼8 min for training, representing an approximate 80% reduction compared with ∼41 min for BI-S2V-DREME. Similar reductions were observed at other epoch settings. Additionally, for S2V-DREME, the average real-time inference latency from two orthogonal slices to a reconstructed 3D volume was 24.0 ± 0.3 ms, including motion encoding, DVF computation, and volume warping.

**Figure 13.**
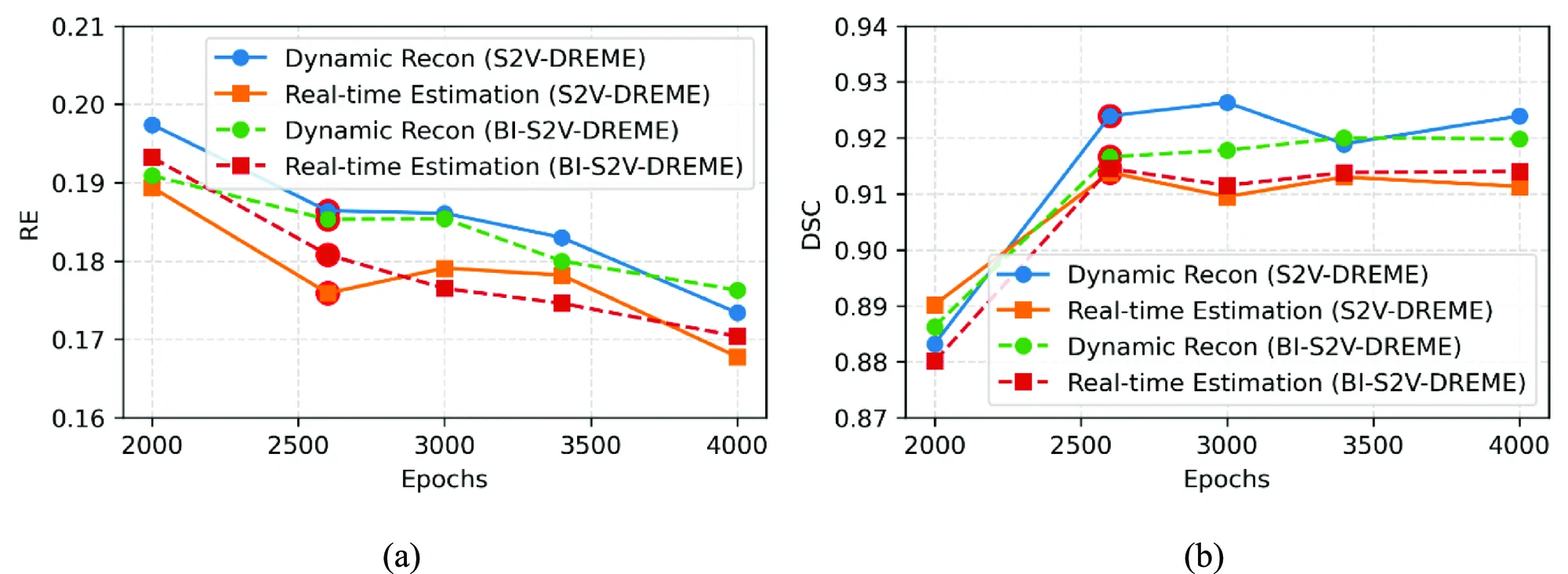
Quantitative performance of S2V-DREME and BI-S2V-DREME in terms of (a) RE and (b) DSC under different epoch settings on the XCAT dataset. Results are reported for both dynamic reconstruction and real-time motion/image estimation tasks.

Following the training time comparison in figure 12, figure 13 compared the quantitative performance of S2V-DREME and BI-S2V-DREME in terms of RE and DSC for dynamic reconstruction and real-time motion estimation under different training epoch settings. As the number of training epochs increased, RE showed a decreasing trend, while DSC increased accordingly, with performance gains becoming less pronounced at higher epoch settings. These results indicated that S2V-DREME achieved comparable or improved accuracy compared with BI-S2V-DREME while requiring substantially less training time. Notably, the RE and DSC values achieved at 2600 epochs were already close to those obtained under higher epoch settings for both dynamic reconstruction and real-time motion/image estimation. Therefore, 2600 epochs were adopted in the subsequent experiments as the default setting. The same epoch setting was used for the physical phantom study. For the human study, 3400 epochs were used ($L = 3,{\text{ }}{N_a} = 1000,{\text{ }}{N_{b1,{\text{ }}L}} = 200$, ${N_{b2,{\text{ }}L}}$ = 600), taking ∼11 min to train S2V-DREME and ∼25 ms for real-time inference on an NVIDIA RTX 4090 GPU.

### 3.5. Hyperparameter analysis

Quantitative results using different slice numbers sampled per location for S2V-DREME training are summarized in table 4 for both dynamic reconstruction and real-time motion/image estimation. As the number of slices per position increased, performance generally improved across most evaluation metrics. The most notable performance gains were observed when increasing the sampled slice number per location from relatively low values (5, 10, and 15), whereas further improvements became marginal at higher values (20, 25, and 30). A similar trend was observed for real-time motion/image estimation accuracy. When 20 or more slices per position were used, the quantitative results tended to stabilize across all metrics for both tasks; therefore, 20 slices per position were selected as the default setting in the subsequent experiments.

**Table 4.** Hyperparameter analysis of the number of slices per position for dynamic reconstruction and real-time motion/image estimation accuracy across five motion scenarios on the XCAT dataset. Dynamic reconstruction accuracy was evaluated on 1,000 training slice pairs (edge locations excluded), and real-time motion/image estimation was based on 300 slice pairs using the trained model. The arrows point to the direction of improved accuracy.

Slice No.	Dynamic reconstruction					Real-time motion/image estimation				
	SSIM↑	RE↓	Dice↑	HD95↓	COME↓	SSIM↑	RE↓	Dice↑	HD95↓	COME↓
5	0.79 ± 0.06	0.23 ± 0.06	0.87 ± 0.13	2.56 ± 2.70	1.75 ± 0.48	0.80 ± 0.02	0.20 ± 0.02	0.87 ± 0.05	2.43 ± 0.93	1.98 ± 0.48
10	0.80 ± 0.07	0.21 ± 0.07	0.89 ± 0.15	1.98 ± 3.07	1.41 ± 0.35	0.82 ± 0.01	0.18 ± 0.01	0.89 ± 0.02	1.89 ± 0.59	1.59 ± 0.64
15	0.81 ± 0.07	0.19 ± 0.07	0.90 ± 0.14	1.77 ± 2.78	1.22 ± 0.31	0.83 ± 0.01	0.17 ± 0.01	0.90 ± 0.02	1.88 ± 0.45	1.07 ± 0.45
20	0.83 ± 0.02	0.18 ± 0.02	0.92 ± 0.03	1.64 ± 0.78	0.98 ± 0.43	0.83 ± 0.02	0.17 ± 0.02	0.91 ± 0.02	1.55 ± 0.87	0.99 ± 0.73
25	0.83 ± 0.07	0.17 ± 0.07	0.91 ± 0.14	1.56 ± 3.05	0.96 ± 0.27	0.84 ± 0.02	0.16 ± 0.01	0.91 ± 0.02	1.50 ± 0.41	0.98 ± 0.60
30	0.83 ± 0.08	0.17 ± 0.08	0.92 ± 0.18	1.50 ± 4.02	0.95 ± 0.54	0.84 ± 0.02	0.16 ± 0.01	0.92 ± 0.01	1.49 ± 0.25	0.98 ± 0.54

As shown in table 5, varying the control-point spacing resulted in relatively small performance differences for both dynamic reconstruction and real-time motion/image estimation tasks. Across the three evaluated settings, all metrics (SSIM, RE, Dice, HD95, and COME) exhibited minor variations, and no specific spacing consistently outperformed the others. These findings indicated that the SINR-based MBC generator was not highly sensitive to the choice of control-point spacing. Thus, we used (5, 3) as the default for the XCAT study. The same setting was applied to physical phantom study. For human study, we used (7, 5, 3), adding an additional level to account for more complicated anatomy/motion features.

**Table 5.** Hyperparameter analysis of the control-point spacing in SINR-based MBC generator for dynamic reconstruction and real-time motion estimation performance across five motion scenarios on the XCAT dataset. Dynamic reconstruction accuracy was evaluated with 1,000 slice pairs (edge locations excluded), and real-time motion estimation was based on 300 slice pairs using the trained model. The arrows point to the direction of improved accuracy.

Tasks	Control-point spacing	SSIM↑	RE↓	Dice↑	HD95(mm)↓	COME(mm)↓
Dynamic Reconstruction	(7, 5)	0.83 ± 0.02	0.18 ± 0.05	0.91 ± 0.10	1.63 ± 0.89	1.04 ± 0.58
	(5, 3)	0.83 ± 0.02	0.18 ± 0.02	0.92 ± 0.03	1.64 ± 0.78	0.98 ± 0.43
	(3, 2)	0.84 ± 0.05	0.18 ± 0.05	0.92 ± 0.13	1.65 ± 1.42	0.99 ± 0.53
Real-time Motion/Image Estimation	(7, 5)	0.83 ± 0.02	0.18 ± 0.03	0.91 ± 0.04	1.56 ± 0.81	1.00 ± 0.88
	(5, 3)	0.83 ± 0.02	0.17 ± 0.02	0.91 ± 0.02	1.55 ± 0.87	0.99 ± 0.73
	(3, 2)	0.84 ± 0.02	0.17 ± 0.05	0.91 ± 0.05	1.56 ± 1.02	1.00 ± 0.73

### 3.6. Evaluation of alternative acquisition strategies for dark-band artifact mitigation

**Figure 14.**
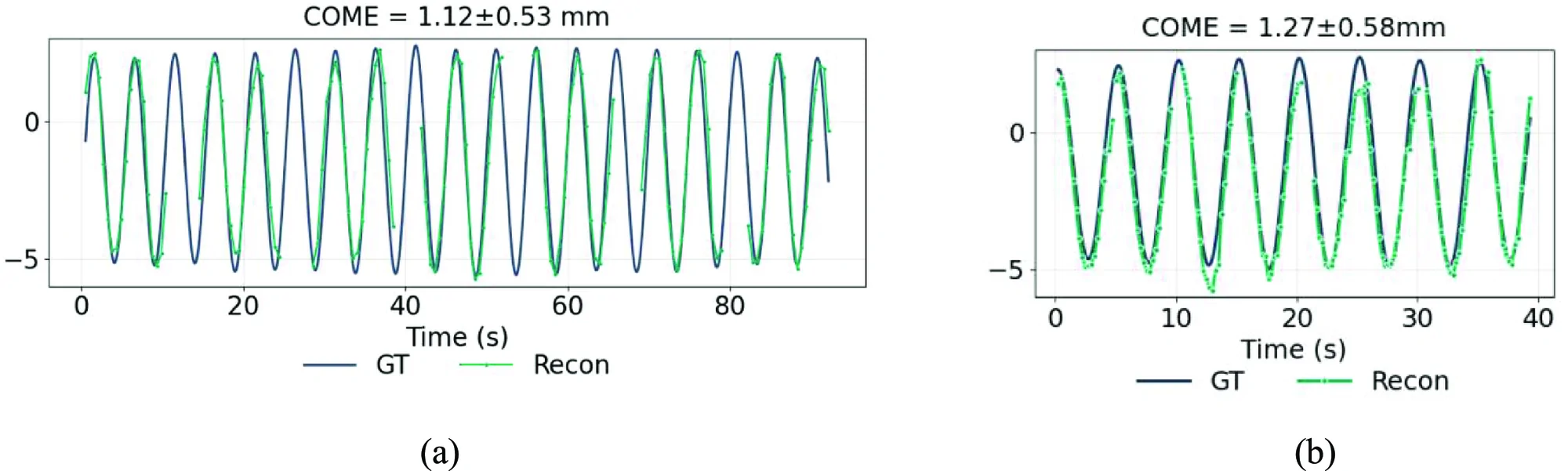
Comparison of reconstructed and ground-truth motion trajectories obtained from dynamic reconstruction using physical phantom measurements (motion scenario P3). (a) Ortho-view BFFE acquisition. (b) Single-view SS-TSE acquisition using the sagittal view.

**Figure 15.**
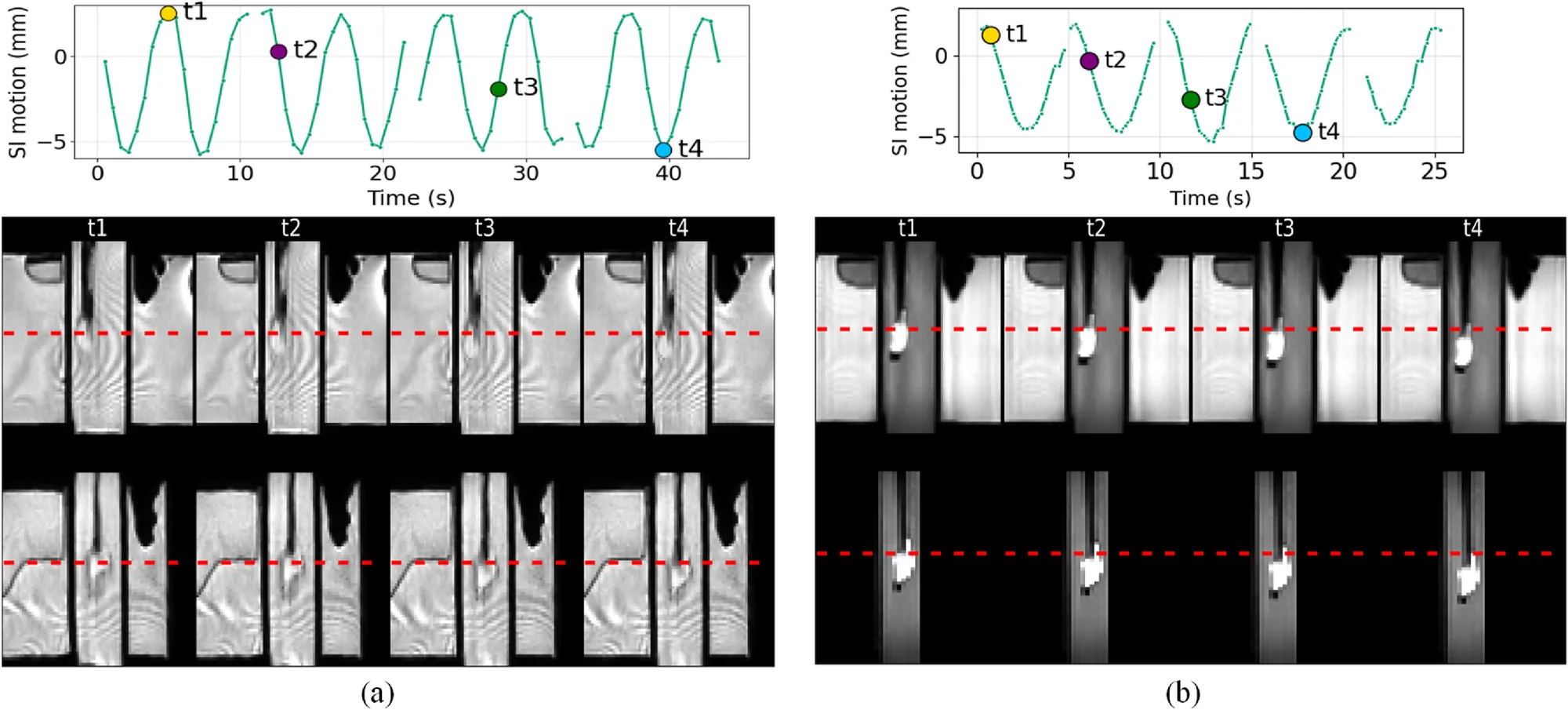
Time-resolved dynamic images of the phantom studies. (a) Ortho-view reconstruction using sagittal and coronal BFFE acquisitions. (b) Single-view reconstruction using only the sagittal SS-TSE acquisition. The first row presents the solved tumor center-of-mass trajectory along the SI direction. The labeled dots (t1–t4) indicate the selected time points for image visualization. The subsequent rows show the reconstructed MR images in the sagittal and coronal views.

**Figure 16.**
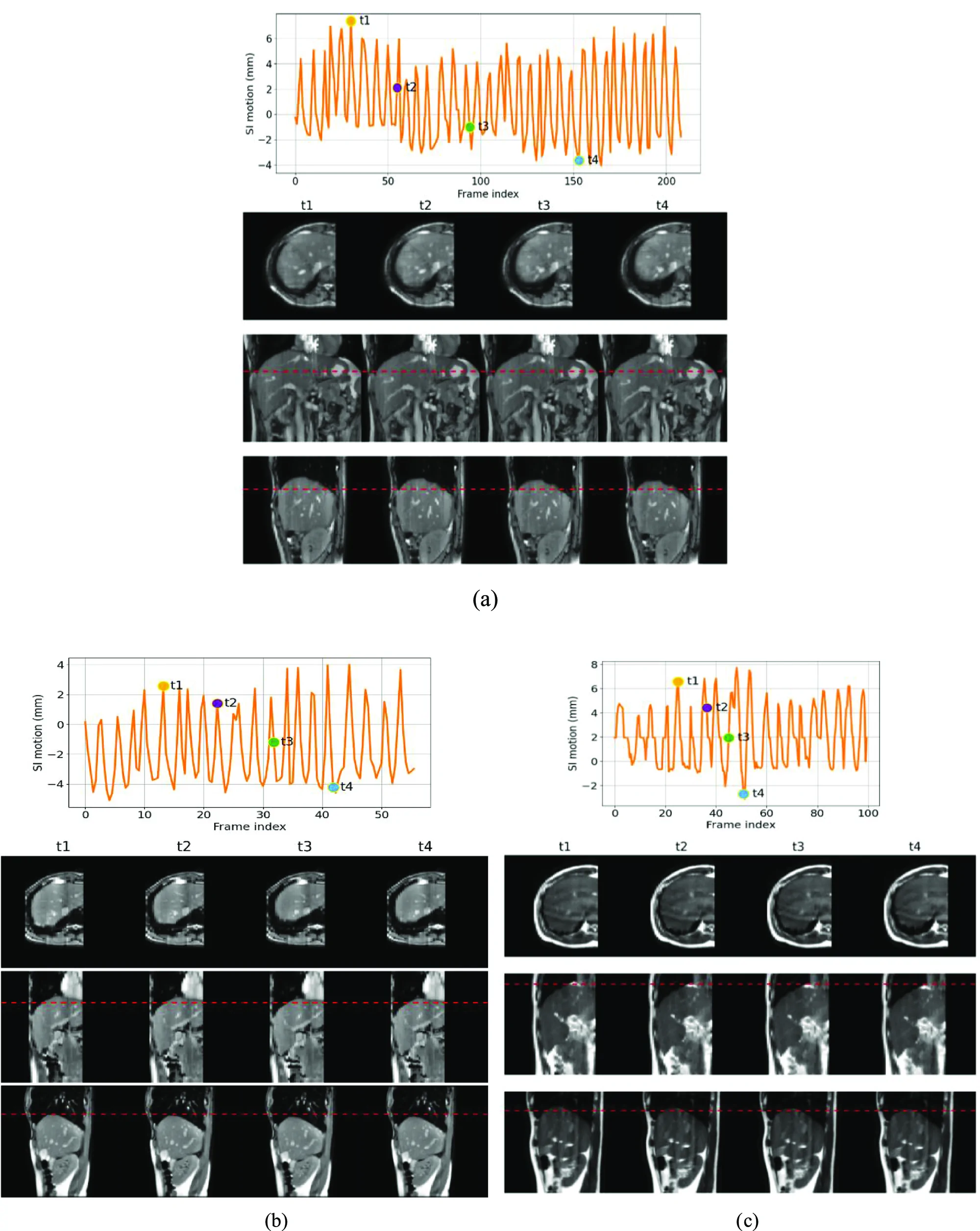
Time-resolved dynamic MR images of the volunteer study. (a) Reconstruction using ortho-view (sagittal and coronal) BFFE acquisitions. (b) Reconstruction using single-view multi-slice (sagittal) BFFE acquisition. (c) Reconstruction using single-view (sagittal) SS-TSE acquisition. For each panel, the first row shows the estimated liver center-of-mass trajectory along the SI direction. The subsequent rows display the reconstructed MR images in the axial, coronal, and sagittal views at four representative time points (*t*1–*t*4). The acquisition field of view was focused on the liver region rather than the entire abdomen.

In this section, we evaluated three acquisition strategies for dark-band artifact mitigation: orthogonal-view BFFE, single-view multi-slice BFFE, and single-view SS-TSE (section 2.7). The resulting target COMEs from orthogonal-view BFFE and single-view SS-TSE acquisitions in the phantom study are presented in figure 14. The corresponding time-resolved dynamic reconstruction results for the phantom and volunteer studies are shown in figures 15 and 16, respectively.

Figure 14 presents the reconstructed motion trajectories obtained using orthogonal-view BFFE and single-view SS-TSE acquisitions under the same physical phantom motion scenario. Compared with the orthogonal-view SS-TSE results in figure 6, the orthogonal-view BFFE acquisition in figure 14(a) achieved a similar COME (1.12 ± 0.53 mm vs 1.17 ± 0.50 mm). In addition, compared with the orthogonal-view SS-TSE acquisition (1.17 ± 0.50 mm), the single-view SS-TSE results showed a slightly higher COME (1.27 ± 0.58 mm).

Figures 15 and 16 show the time-resolved dynamic reconstruction results for the phantom and volunteer studies, respectively. Some aliasing artifacts from BFFE acquisitions, especially in the phantom study (figure 15(a)), can be seen. Compared with the orthogonal-view SS-TSE results in figure 9, the orthogonal-view BFFE results in figure 16(a) showed less pronounced dark-band artifacts. Similarly, single-view multi-slice BFFE-based reconstructions in figure 16(b) and single-view SS-TSE-based reconstructions in figure 16(c) showed less pronounced dark-band artifacts. Overall, the BFFE-based reconstructions in figures 16(a) and (b) exhibited better visual image quality than their SS-TSE counterparts. The orthogonal-view BFFE acquisition incorporated complementary high-in-plane-resolution information from the coronal view, yielding reconstructions of better resolution in the sagittal direction than the single-view BFFE-based reconstructions.

### 3.7. Generalization and robustness enhancement with motion augmentation

**Figure 17.**
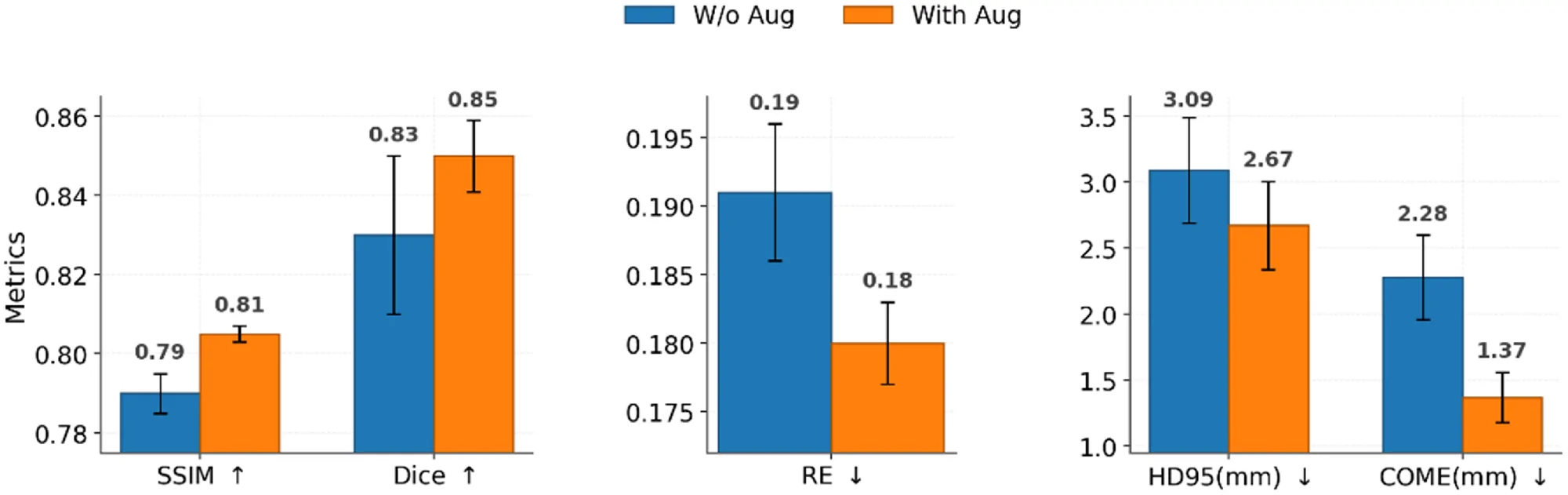
Quantitative comparison of real-time tumor motion estimation performance on the XCAT dataset with and without the motion score augmentation strategy. The model was trained separately on each motion pattern (dynamic reconstruction) and then evaluated on Motion 1–5 (real-time motion inference). The bars represent the average results over all experiments.

**Figure 18.**
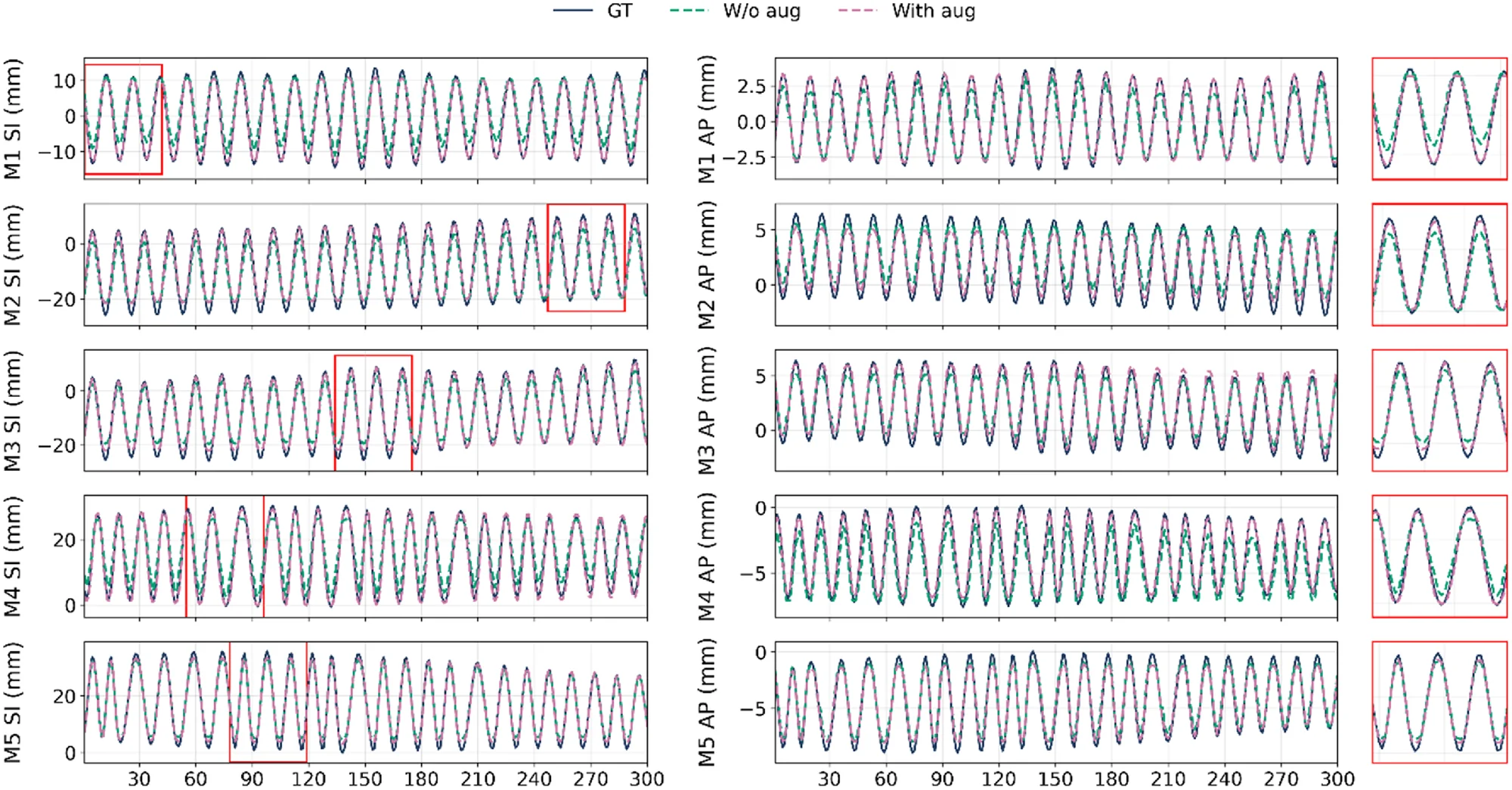
Real-time tumor motion estimation curves with and without motion augmentation on the XCAT dataset for five motion patterns (M1–M5) in the SI (left) and AP (right) directions. For the plots, the model was trained on Motion 5 and evaluated on Motion 1–5. A zoomed-in local region of the SI trajectory is shown on the right to facilitate visualization of local differences.

To evaluate the effect of motion score augmentation on real-time motion estimation under unseen respiratory patterns, S2V-DREME was trained separately on each XCAT motion with and without augmentation and tested on all five motion scenarios. Figure 17 summarizes the quantitative results averaged over all experiments. Figure 18 presents real-time motion estimation curves in the SI and AP directions for all motion patterns, comparing the predicted trajectories with and without augmentation against the GT.

Quantitative results in figure 17 show that motion augmentation improved real-time motion estimation performance on unseen motion patterns, reducing COME from 2.28 ± 0.32 mm to 1.37 ± 0.19 mm and increasing the Dice coefficient from 0.83 ± 0.02–0.85 ± 0.01 compared with the model trained without augmentation. Additional improvements were also observed in SSIM, RE, and HD95 metrics. Figure 18 presents real-time motion estimation curves in the SI and AP directions for all five motion patterns. The model trained with motion augmentation generated trajectories that more closely matched the GT and exhibited reduced deviations across different motion patterns. These results indicate that the motion augmentation strategy enhances the robustness of the motion encoder when the testing motion patterns differ from those observed during training.

## 4. Discussion

This work proposed a slice-to-volume time-resolved MRI framework that enables both dynamic reconstruction and real-time motion/image estimation from 2D cine MR slices. In contrast to conventional slice-driven 4D-MRI techniques, which rely on motion sorting and are thus sensitive to respiratory irregularities, S2V-DREME reconstructs time-resolved 3D MRIs from each acquired slice, avoiding motion sorting and capturing both regular and irregular motion trajectories. From a pre-treatment scan, S2V-DREME jointly reconstructs a reference patient anatomy and learns a data-driven, patient-specific motion model within a ‘one-shot’ framework, enabling it to capture the most up-to-date anatomy and motion patterns with minimized generalizability concerns. In addition, the design of S2V-DREME is motivated by the computational burden of the original DREME-MR framework, which requires repeated transformations between the image and k-space domains to enforce data consistency during training. By directly optimizing the objective function in the 2D-slice-based image domain, S2V-DREME avoids repeated domain transformations and simplifies the reconstruction pipeline. Beyond computational efficiency, slice-based reconstruction from 2D acquisitions facilitates clinical deployment and offers flexibility in the choice of T1-, T2-, or mixed T2/T1-weighted sequences (e.g. BFFE (Scheffler and Lehnhardt 2003)). In contrast, many k-space-driven time-resolved reconstruction frameworks have been implemented with gradient-echo readouts to maintain high temporal resolution, which limits their ability to achieve genuine T2-weighted contrast. This constraint is particularly relevant in abdominal imaging, where T2-weighted contrast can improve target visualization and localization. In this study, we demonstrate the feasibility of using T2-weighted SS-TSE cine imaging for time-resolved 3D MRI reconstruction. To further enhance the efficiency of the overall S2V-DREME framework, a SINR-based MBC generator is introduced to accelerate the learning of MBCs. In addition, a FiLM-based motion encoder is incorporated to enhance motion-dependent feature modulation, improving reconstruction accuracy without increasing inference cost. These architectural designs allow S2V-DREME to preserve spatial fidelity and temporal resolution under highly sparse slice sampling conditions, making the framework suitable for both reconstruction-stage optimization and real-time inference while addressing the trade-off among accuracy, efficiency, and spatiotemporal resolution. While current reconstructed volumes have lower spatial resolution and image quality than conventional diagnostic MRI, the primary objective of this study is not to generate diagnostic-quality images, but to demonstrate the feasibility of reconstructing time-resolved volumetric anatomy from rapidly acquired sparse 2D images for motion monitoring and potential motion management applications. With this balance, the achieved image quality and spatial resolution can support the intended application of S2V-DREME in time-resolved volumetric motion monitoring and potential motion management.

The ablation and hyperparameter analyses validate the key properties of the proposed framework. The XCAT results demonstrate that a two-slice configuration outperformed the single-slice setting (table 3), as orthogonal slices provide complementary motion information along the SI and AP directions. This complementary sampling improves sensitivity to multi-directional respiratory motion, enabling more accurate motion estimation and tumor tracking, though dark band artifacts from clinical acquisitions may occur due to constant view switching (orthogonal-view SS-TSE acquisition, for instance). Furthermore, incorporating slice location information explicitly enhances the model’s ability to disambiguate anatomy and motion states, leading to additional performance gains. In addition, the hyperparameter analysis demonstrated that satisfactory reconstruction quality could be achieved with limited data acquisition (approximately 7 s per slice location for XCAT, derived from 20 slice pairs at 0.35 s per pair). Finally, the analysis of the control-point grid size for MBC generation indicates that the proposed framework is relatively insensitive to this parameter, although further studies, especially on patients, are warranted.

The XCAT results demonstrate that S2V-DREME consistently outperformed conventional 4D-MRI baselines in both reconstruction quality and motion tracking accuracy. The comparable performance observed between dynamic reconstruction and real-time motion/image estimation suggests that the learned model may generalize beyond the training conditions, which is important for real-time deployment. The physical phantom experiments further confirmed stable intra-measurement dynamic reconstruction and cross-measurement real-time testing under different motion conditions, while the human studies produced anatomically consistent dynamic MR images under realistic respiratory motion. The ablation and hyperparameter analyses further validated the benefit of slice-location modulation, SINR-based motion basis component generation, and the robustness of the proposed framework to key parameter choices. In addition, motion augmentation experiments demonstrated that perturbing the learned motion scores improved the robustness of the motion encoder, enabling more accurate real-time motion estimation when testing motion patterns differed from those observed during training.

Despite the demonstrated feasibility and efficiency of S2V-DREME in dynamic reconstruction and real-time motion/image estimation, several limitations warrant further investigation. First, the current framework relies on the implicit neural representation to model the reference anatomy, and all reconstructed volumes are obtained by warping this reference using the estimated deformation fields. Consequently, the accuracy of the reference representation plays a critical role in overall reconstruction quality. Exploring alternative or more expressive representations for reference anatomy modeling may further improve performance (Xie *et al* 2025). Second, dark-band artifacts remain a challenge in orthogonal interleaved cine acquisitions using SS-TSE, as shown in figures 7–9, primarily due to disturbances of the magnetization steady state and slice history. To mitigate this issue, we further evaluated orthogonal-view BFFE, single-view multi-slice BFFE, and single-view SS-TSE acquisitions, and all showed reduced dark-band artifacts compared with orthogonal-view SS-TSE. Residual artifacts, including aliasing artifacts of BFFE acquisitions (figure 15(a)), can still be seen. Future work may explore additional strategies to mitigate artifacts, such as in-slice band artifact correction (Bangerter *et al* 2004) prior to reconstruction, using pre-acquired high-resolution volumes to improve the learned reference volume quality (Zhao *et al* 2020), or deep learning-based reconstruction methods with artifact suppression (Hammernik *et al* 2018). Validation across more subjects and motion scenarios is also needed to assess and evaluate the image artifacts and their impacts on image quality and motion estimation accuracy, under different image acquisition and artifact suppression strategies. In addition, to further improve the reconstruction accuracy from a single-view input, the single-view slices could potentially be combined with additional signals, such as breathing-volume-based respiratory surrogates (Tran *et al* 2020) or MR-compatible surface imaging (Li 2022, Shao *et al* 2023), to provide more clues for accurate motion reconstruction and inference. To address the poor resolution along the missing-view direction in single-view acquisitions, additional super-resolution strategies could be used to improve the spatial resolution (Li *et al* 2025a, 2025b). In addition, for the human study, we only qualitatively evaluated the dynamic reconstruction results due to the limited acquisition data and the lack of GT for reference. In future human studies, we can acquire two sets of data at different time points for cross-set tests, by using the dynamic reconstruction results of each set as a reference for the quantitative evaluation of real-time motion/image estimation. Finally, both the original DREME-MR framework and the proposed S2V-DREME adopt relatively complex training strategies. Further strategies to reduce training complexity while preserving model performance, for instance, by prior-driven model adaptation (Zuo *et al* 2025), should be further explored.

## 5. Conclusions

This work presented S2V-DREME, a slice-to-volume motion-resolved MRI framework for dynamic volumetric reconstruction and real-time motion/image estimation from 2D cine slices. By combining a novel step-and-shoot acquisition protocol with motion-compensated ‘one-shot’ learning, S2V-DREME leverages pre-treatment slice-based imaging to learn patient-specific anatomy and motion patterns and enables continuous intra-treatment real-time volumetric motion monitoring using 2D slice acquisitions. The results demonstrate accurate and temporally consistent motion estimation, highlighting its potential for rapid volumetric imaging and real-time MR-guided adaptive radiotherapy under constrained acquisition conditions.

## Data Availability

The data supporting the findings of this study are available from the corresponding author upon reasonable request. Volunteer imaging data are not publicly available due to privacy and ethical restrictions. Supplementary Materials available at https://doi.org/10.1088/1361-6560/ae9d13/data1. Supplementary Video 1 available at https://doi.org/10.1088/1361-6560/ae9d13/data2. Supplementary Video 2 available at https://doi.org/10.1088/1361-6560/ae9d13/data3. Supplementary Video 3 available at https://doi.org/10.1088/1361-6560/ae9d13/data4. Supplementary Video 4 available at https://doi.org/10.1088/1361-6560/ae9d13/data5.
